# Lysine lactylation regulates ATF4-mediated stress responses under glucose starvation in canine hemangiosarcoma

**DOI:** 10.3389/fvets.2026.1734339

**Published:** 2026-02-12

**Authors:** Tamami Suzuki, Kazuki Heishima, Jumpei Yamazaki, Masaya Yamazaki, Ryohei Kinoshita, Sangho Kim, Kenji Hosoya, Yuko Okamatsu-Ogura, Michihito Sasaki, Peng Xu, Qin Yan, Takashi Kimura, Keisuke Aoshima

**Affiliations:** 1 Laboratory of Comparative Pathology, Department of Clinical Sciences, Faculty of Veterinary Medicine, Hokkaido University, Sapporo, Hokkaido, Japan; 2 The United Graduate School of Drug Discovery and Medical Information Sciences, School of Medicine, Gifu University, Gifu, Japan; 3 Institute for Advanced Study, Gifu University, Gifu, Japan; 4 Center for One Medicine Innovative Translational Research (COMIT), Gifu University, Gifu, Japan; 5 Translational Research Unit, Veterinary Teaching Hospital, Faculty of Veterinary Medicine, Hokkaido University, Sapporo, Hokkaido, Japan; 6 Cancer Research Unit, One Health Research Center, Hokkaido University, Sapporo, Hokkaido, Japan; 7 Division of Carcinogenesis, The Cancer Institute, Japanese Foundation for Cancer Research, Tokyo, Japan; 8 Veterinary Teaching Hospital, Faculty of Veterinary Medicine, Hokkaido University, Sapporo, Hokkaido, Japan; 9 Laboratory of Advanced Veterinary Medicine, Department of Clinical Sciences, Faculty of Veterinary Medicine, Hokkaido University, Sapporo, Hokkaido, Japan; 10 Laboratory of Biochemistry, Department of Basic Veterinary Sciences, Faculty of Veterinary Medicine, Hokkaido University, Sapporo, Hokkaido, Japan; 11 Division of Molecular Pathobiology, International Institute for Zoonosis Control, Hokkaido University, Sapporo, Hokkaido, Japan; 12 Department of Pathology, Yale School of Medicine, New Haven, CT, United States

**Keywords:** dog, glucose, hemangiosarcoma, histone lactylation, stress response, tumor metabolism

## Abstract

Excess lactate is produced in tumor cells by aerobic glycolysis and regulates gene expressions by histone lactylation. However, how histone lactylation functions under glucose-limited conditions remains unknown. Here, we show that lysine lactylation redistributes to transcription start sites (TSSs) during glucose deprivation, thereby altering biological behaviors in canine hemangiosarcoma (HSA) cells. Glucose deprivation significantly decreased global histone lactylation levels, while lactylation peaks were enriched at TSSs of ATF4-regulated stress-response, asparagine-synthesis and immune-related genes. Stress-response gene expressions were upregulated, and ATF4 polyclonal knockout abrogated this activation. [U-^13^C]glutamine tracing demonstrated that HSA cells synthesized asparagine from glutamine when glucose was scarce, and asparagine supplementation modestly activated cell proliferation. In HSA patient tissues, H3K18la levels were heterogeneous, and M2-like macrophages preferentially infiltrated tumor regions showing low histone lactylation levels. These findings demonstrate that lysine lactylation regulates transcription that supports tumor cell survival and fosters a pro-tumor microenvironment even under glucose-limited conditions.

## Introduction

Tumor cells exhibit metabolic profiles distinct from those of normal cells, through which they can generate sufficient ATP and nucleotide building blocks to survive in harsh environments such as hypoxia and nutrient-poor conditions (1, 2). Even under aerobic conditions, tumor cells upregulate glycolysis on top of oxidative phosphorylation (OXPHOS), which results in excess lactate production (3). Glutamine, a nutrient used to supply nucleotides and intermediate metabolites via anaplerosis, also contributes to lactate production through glutaminolysis (4–6). Recent studies have shown that lactate is not merely a waste product, rather it is an important substrate for an epigenetic mark, histone lysine lactylation (7). In macrophages, histone lactylation upregulates reparative genes such as *Arg1*, and its levels are reduced by glycolysis inhibition and rescued by exogenous lactate supplementation (8). In tumors, histone lactylation has been implicated in promoting immunosuppression and tumor progression (9–13). Despite the increasing number of histone lactylation studies, most have been conducted under high-lactate conditions. Little is known about the roles of histone lactylation under low-lactate conditions.

Hemangiosarcoma (HSA) is a malignant tumor of vascular endothelial cells. In dogs, HSA accounts for 1.3–2.8% of all canine tumors and often arises in the spleen, liver and right atrium of the heart (14–17). One study reported 4,997 HSA cases among 2.2 million dogs (2,271 per million) (18). Given that HSA is highly invasive, rapid metastasis and tumor rupture occur frequently and contribute to the poor prognosis (19). While surgery and doxorubicin-based chemotherapy remain the standard of care, median survival times are only about five to six months and the one-year survival rate for dogs is less than 16% (20). HSA shares notable similarities with human angiosarcoma in morphological features, genetic mutations (e.g., *TP53*, *PIK3CA*, *ATRX*), and aggressive clinical behavior (21–23). However, human angiosarcoma is extremely rare. Several studies have estimated an incidence of ~ 3 cases and ~1.5–2.6 cases per million people in the United States and Europe, respectively (24, 25). Due to this rarity, canine HSA is recognized as a valuable spontaneous model for studying angiosarcoma biology and evaluating novel therapeutics (22, 26). Normal endothelial cells rely almost exclusively on aerobic glycolysis to generate ATP and therefore produce substantial lactate. Recent studies show that histone lactylation in endothelial cells promotes angiogenesis and endothelial-to-mesenchymal transition (27–29). Given that HSA cells are neoplastic endothelial cells, we hypothesized that histone lactylation modulates gene expression in HSA and may contribute to its pathogenesis.

In this study, we established canine HSA cell lines and patient-derived xenograft (PDX) models from dog patients and examined the role of histone lactylation under glucose-deprived conditions. Glucose restriction reduced global histone lactylation levels, while lactylation peaks were redistributed to the transcription-start sites (TSSs) of ATF4-regulated stress-response genes, asparagine synthesis genes, and immune-related genes. TSSs of stress-response genes were co-occupied by RNA polymerase II phosphorylated at serine 5 (RNAPII-Ser5P), and the associated genes showed increased transcription, suggesting that these lactylation marks activated transcription. [U-^13^C]glutamine tracing in HU-HSA-3 cells revealed *de novo* asparagine synthesis from glutamine under glucose-deprived conditions, and asparagine supplementation modestly activated cell proliferation *in vitro*. In clinical HSA tissues, H3K18la signals were heterogeneous, but tumor regions with low H3K18la signals accumulated M2-like macrophages. Consistently, HSA cells attracted macrophages and promoted their differentiation toward the M2-like state, suggesting that HSA tumor cells establish a pro-tumor microenvironment in low-lactylation regions. Together, our data show that lysine lactylation, possibly histone lactylation, persists even under glucose-deprived conditions, suggesting that tumor cells use lactate to regulate transcription under nutrient-poor conditions.

## Materials and methods

### Reagents, kits, and instruments

All reagents, kits, and instruments used in this study are summarized in Tables 1, 2 along with their manufacturer information and catalog numbers.

**Table 1:** Antibodies for Immunohistochemistry (IHC), western blotting (WB), and CUT&Tag (C&T).

Antibody	Supplier	Catalog number	Clone name	Dilution
anti-CD31	Abcam	ab134168	EP3095	IHC 1:200 WB 1:1000
anti-Von Willebrand Factor	Agilent	A0082		IHC 1:500 WB 1:1000
anti-VEGF Receptor 2 (KDR)	Abcam	ab2349		WB 1:1000
anti-L-Lactyl-Histone H3 Lys18 rabbit monoclonal antibody	PTM Biolabs	PTM-1406RM		IHC 1:250 WB 1:1000
anti-Iba1	Fujifilm wako	019–19,741		IHC 1:500
anti-CD3	Agilent technologies	IR503		IHC ready to use
anti-CD204	Medicinal Chemistry Pharmaceutical Co., Ltd.	KT022	SRA-E5	IHC 1:200
anti-L-Lactyllysine	PTM Biolabs	PTM-1401RM	9H1L6	C&T 1:50 IHC 1:200
anti-Tri-Methyl-Histone H3 (Lys4)	Cell signaling technology	9,751	C42D8	C&T 1:50
anti-Acetyl-Histone H3 (Lys27)	Cell signaling technology	8,173	D5E4	C&T 1:100
anti-Phospho-Rpb1 CTD (Ser5)	Cell signaling technology	13,523	D9N5I	C&T 1:50
anti-L-Lactyl-Histone H4 (Lys5) Rabbit mAb	PTM biolabs	PTM-1407RM		WB 1:1000
anti-Acetylated Histone H3	Active motif	39,040		WB 1:5000
anti-Acetylated Histone H4	Santa Cruz Biotechnology, Inc.	sc-377520	E-5	WB 1:500
anti-Actin	Sigma-Aldrich	MAB1501	C4	WB 1:10000
anti-Histone H3	MAB institute	MABI0001-20	CMA301	WB 1:25000
anti-ATF-4	Santa Cruz Biotechnology, Inc.	sc-390063	B-3	WB 1:1000
anti-Asparagine synthethase	Santa Cruz Biotechnology, Inc.	sc-365809	G-10	WB 1:1000
anti-Glut1	Santa Cruz Biotechnology, Inc.	sc-377228	A-4	WB 1:1000
Goat anti-Mouse IgG (H + L)	Thermo Fisher Scientific	G21040		WB 1:10000
Goat anti-Rabbit IgG (H + L)	Thermo Fisher Scientific	G21234		WB 1:10000

**Table 2:** Reagents and instruments.

Reagent		
Product name	Supplier	Catalog number
Canine genotypes panel 2.1	Thermo Fisher Scientific, MA, USA	F864S
Dulbecco’s Modified Eagle Medium with High Glucose	Fujifilm Wako Pure Chemical Industries, Osaka, Japan	044-29765
Dulbecco’s Modified Eagle Medium with no Glucose	Fujifilm Wako Pure Chemical Industries	042-32255
Dulbecco’s Modified Eagle Medium with no glutamine	Fujifilm Wako Pure Chemical Industries	045-30285
Fetal bovine serum	Gibco, NY, USA	10270-106
penicillin–streptomycin solution	Fujifilm Wako Pure Chemical Industries	168-23191
EASYstrainer (70 μm)	Greiner Bio-One, Kremsmünster, Austria	542070
TaKaRa BCA Protein Assay Kit	Takara Bio, Kusatsu, Japan	T9300A
Immobilon-P transfer membranes	Merck Millipore, MA, USA	IPVH00010
Can Get Signal Solution	TOYOBO, Osaka, Japan	NKB-101
Immobilon Western Chemiluminescent HRP substrate	Merck Millipore	WBKLS0500
10% normal goat serum	Nichirei biosciences, Tokyo, Japan	426042
goat anti-rabbit IgG conjugated peroxidase	Nichirei biosciences	414341
3.3′-diaminobenzidine	Dojindo, Kumamoto, Japan	349-00903
goat anti-rabbit IgG conjugated alkaline phosphatase	Nichirei biosciences	414251
goat anti-mouse IgG conjugated alkaline phosphatase	Nichirei biosciences	414241
New Fuchsin solution	Nichirei biosciences	415161F
12-well plate	Greiner Bio-One	665180
Asparagine	MP Biomedicals, CA, USA	590-20432
Proline	Fujifilm Wako Pure Chemical Industries	163-04601
0.25 w/v% Trypsin-1 mmol/L EDTA 4Na Solution with Phenol Red	Fujifilm Wako Pure Chemical Industries	201-16945
Lipofectamine 3,000	Thermo Fisher Scientific	L3000015
0.45 μm pore filter	Sartorius, Göttingen, Germany	S7598FXOSK
Domitor (medetomidine)	ZENOAQ, Tokyo, Japan	N/A
Dormicum (midazolam)	Maruishi Pharmaceutical Co., Ltd. Osaka, Japan	211-762100
Vetorphale (butorphanol)	Meiji Seika Pharma Co., Ltd. Tokyo, Japan	N/A
Atipame (atipamezole)	Kyoritsu Seiyaku Corporation, Tokyo, Japan	N/A
Seahorse XFp Cell Culture Miniplate	Agilent Technology, CA, USA	103025-100
Seahorse XF base medium without phenol red	Agilent Technology	103335-100
L-glutamine	Fujifilm Wako Pure Chemical Industries	073-05391
D(+)-glucose	Fujifilm Wako Pure Chemical Industries,	047-31161
ATP rate assay Kit	Agilent Technology	103591-100
mitochondrial oxidation assay Kit	Agilent Technology	103270-100
Hoechst 33342	Dojindo, Kumamoto, Japan	346-07951
CUT&Tag Assay Kit	Cell Signaling Technology, MA, USA	77752
PCR Master Mix	New England Biolabs, MA, USA	M0541S
AMpure	Beckman Coulter, CA, USA	BC-A63880
dsDNA high sensitivity kit	Invitrogen, MA, USA	Q33230
D5000 High sensitivity kit	Agilent Technology	5067-5089
NucleoSpin RNA isolation kit	Macherey-Nagel GmbH & Co. Düren, Germany	740955.5
TriPure Isolation Reagent	Roche, Basel, Switzerland	11667157001
Primescript II 1st strand cDNA Synthesis Kit	Takara Bio	6210
KAPA SYBR FAST qPCR Kit Master Mix (2×) ABI Prism	KAPA Biosystems, MA, USA	KK4605
ThinCert Cell Culture Inserts	Greiner Bio-One	657630
0.2 μm pore filter	Sartorius	S7597FXOSK
Dead Cell Removal Kit	Miltenyi Biotec, Bergisch Gladbach, Germany	130-090-101
Chromium Next GEM Single Cell 3′ Kit v3.1, 4 rxns	10x Genomics, CA, USA	PN-1000269
Chromium Next GEM Chip G Single Cell Kit, 16 rxns	10x Genomics	PN-1000127
Dual Index Kit TT Set A, 96 rxns	10x Genomics	PN-1000215
High Sensitivity DNA Kit	Agilent Technology	5067-4727
Tissue-Tek^®^ O.C.T. Compound	Sakura Finetek Japan Co., Ltd., Tokyo, Japan	4583
13C5 L-glutamine	Fujifilm Wako Pure Chemical Industries, Osaka, Japan	CLM-1822-H-0.1
erythritol	Tokyo Chemical Industry Co., Ltd., Tokyo, Japan	E0021
Ultracentrifugation for 5 kDa cut-off filter	Human Metabolome Technology, Tsuruoka, Japan	UFC3LCCNB_HMT
96 well cell culture plates	Greiner Bio-One	655180
Cell Counting Kit-8	Dojindo	343-07623
Eukitt	ORSAtec, Bobingen, Germany	6.00.01.0001.06.01.EN
BRANSON Sonifier 450	Branson Ultrasonics Corporation, CT, USA	

Instruments		
Product name	Supplier	Catalog number
Image Quant LAS 4000 mini luminescent image analyzer	Cytiva, MA, USA	
NanoZoomer 2.0-RS	Hamamatsu Photonics, Hamamatsu, Japan	
CellDrop BF	DeNovix, DE, USA	
Seahorse XFp Analyzer	Agilent Technology	
EVOS FL	Thermo Fisher Scientific	
Qubit	Invitrogen, MA, USA	
TapeStation	Agilent Technology	
StepOne Real-time PCR system	Thermo Fisher Scientific	
BX-41	Olympus, Tokyo, Japan	
Chromium controller	10x Genomics	
MTP-320	Corona Electric Co., Ltd., Ibaraki, Japan	
Tissue-Tek VIP5 Jr.	Sakura Finetek Japan Co., Ltd	

### Establishment and characterization of canine HSA cell lines and PDX models

Canine HSA cell lines (HU-HSA-2 and HU-HSA-3) and PDX models (HU-HSAPDX-1, HU-HSAPDX-2, and HU-HSAPDX-3) were established from fresh hemangiosarcoma tissues obtained from canine patients that underwent splenectomy at Hokkaido University Veterinary Teaching Hospital (HUVTH) with written informed consent from the owners and approval from the HUVTH Ethics Screening Committee (2022–005). All cases were confirmed as hemangiosarcoma by two board-certified veterinary pathologists. Patient information is provided in Table 3. Tumor tissues were used for cell line establishment and PDX development immediately after surgical resection.

**Table 3:** Patient information.

For cell lines and PDX model establishment				
Patient ID	Breed	Age	Sex	Location
HU-HSA-1	Standard poodle	12y3m	M	Spleen
HU-HSA-2	Toy poodle	11y1m	M Cast	Spleen
HU-HSA-3	Flat coated retriever	8y	F Spay	Spleen

For spatial transcriptomics			
Breed	Age	Sex	Location
Yorkshire terrier	13y3m	M Cast	Spleen

For IHC analysis				
Case number	Breed	Age	Sex	Location
1	Great Pyrenees	9y11m	M	Spleen
2	Miniature schnauzer	11y	M	Spleen
3	Golden retriever	11y	M	Spleen
4	Dachshund	7y9m	M Cast	Spleen
5	Golden retriever	7y8m	F Spay	Spleen
6	Miniature schnauzer	12y10m	F Spay	Spleen
7	Labrador retriever	8y	M	Spleen
8	Mix	9y3m	F	Spleen
9	French bulldog	13y1m	M	Spleen
10	Beagle	12y6m	F Spay	Spleen
11	Dachshund	14y5m	M Cast	Spleen
12	French bulldog	7y8m	M Cast	Spleen
13	Jack Russell terrier	9y11m	M Cast	Spleen

F: female, M: male, Spay: spayed, Cast: castrated.

For cell line establishment, tumor tissues were washed with phosphate-buffered saline (PBS) and then mechanically minced into small fragments (~1–2 mm cubes) with sterile scalpels and scissors. Tissue fragments were washed with PBS and then treated with NH₄Cl buffer {8.3% NH_4_Cl and 170 mM Tris–HCl (pH 7.5)} with gentle agitation twice to remove red blood cells (RBC). Following this, tissue fragments were enzymatically digested in Dulbecco’s Modified Eagle Medium (DMEM) containing 3 mg/mL collagenase I for 50 min at 37 °C with intermittent mixing. Then, the digested tissues were homogenized by sequential passage through 18G and 23G needles, followed by filtration through 70 μm cell strainers to remove remaining tissue fragments. The PBS wash and RBC-lysis steps were repeated, and then the isolated cells were seeded and maintained in 10 cm culture dishes with DMEM supplemented with 10% fetal bovine serum (FBS) and penicillin–streptomycin (100 units/mL penicillin, 100 μg/mL streptomycin) (complete DMEM) at 37 °C in a humidified 5% CO₂ incubator. Cells were used for this study after 10 passages by which time the cultured cells appeared homogeneous and proliferated stably. Cell lines were successfully established from HU-HSA-2 and HU-HSA-3; however, we were unable to establish a stable cell line from HU-HSA-1. To validate that the established cell lines were hemangiosarcoma cells, endothelial marker gene (*PECAM1*, *VWF*, *KDR*) and protein expressions (CD31, vWF, KDR) were assessed by reverse transcription quantitative polymerase chain reaction (RT-qPCR) and western blotting. Subcutaneous transplantation of HU-HSA-2 and HU-HSA-3 cells into the both flanks of three 6- to 8-week-old female KSN/Slc mice (Japan SLC, Inc. Shizuoka, Japan) was conducted to evaluate their tumorigenicity and the morphological characteristics of cell-derived tumors. Detailed transplantation procedures are described in the Animal studies section below. Tumors were resected once any mouse reached the predefined endpoint (total tumor volume of 1 cm^3^ per mouse) and were subjected to histopathological analysis. Cell line authentication was performed by short tandem repeat (STR) analysis using the Canine Genotypes Panel 2.1 Kit.

For PDX establishment, splenic tumor tissue fragments (approximately 1 cm^3^) from canine patients were kept on ice in DMEM, processed, and transplanted as described below within 24 h of resection. The tissues were further cut into approximately 2–3 mm^3^ fragments. Two or three 6- to 8-week-old male or female KSN/Slc mice (Japan SLC, Inc., Shizuoka, Japan) were anesthetized with medetomidine (0.3 mg/kg), midazolam (4 mg/kg), and butorphanol (5 mg/kg). A 7-mm skin incision was made on each flank, and one tumor fragment per flank was implanted subcutaneously. Incisions were closed with surgical clips. For postoperative analgesia, meloxicam (0.2 mg/kg) was administered intraperitoneally on the day of surgery and the following day. Surgical clips were removed 1 week after transplantation. Tumors were resected when they reached a volume of 1 cm^3^, re-cut into 2–3 mm^3^ fragments, and transplanted into recipient mice using the same procedure. This passaging procedure was repeated three times, after which the tissues were designated canine HSA PDX models (HU-HSAPDX-1, HU-HSAPDX-2, and HU-HSAPDX-3). Histopathological examination and immunohistochemistry for endothelial markers were performed to confirm that HSA PDX models retained patient tumor features. Authentication was done by STR analysis using the Canine Genotypes Panel 2.1 Kit.

### Cell line and cell culture

Canine HSA cell lines, HU-HSA-2 and HU-HSA-3, were established as described above. Madin-Darby Canine Kidney cells (MDCK) were obtained from American Type Culture Collection. Human embryonic kidney 293 T cells and RAW264 cells were obtained from RIKEN Bioresource Center. Normally, cells were cultured in high-glucose DMEM supplemented with 10% FBS and penicillin–streptomycin at 37 °C with 5% CO_2_. Glucose-free DMEM was used for glucose-starvation experiments, which include extracellular flux analysis, mRNA-seq, Cleavage Under Targets and Tagmentation (CUT&Tag), and isotope-tracing analysis. Glutamine-free DMEM was used for experiments evaluating global histone lactylation levels (western blotting) under glutamine-free conditions. For asparagine or proline supplementation experiments, the FBS concentration was reduced to 1%. Cell culture dishes were coated with 0.1% gelatin when culturing HU-HSA-2 and HU-HSA-3 to improve cell attachment, reduce cell aggregation, and reproducibility. All cell lines used in this study were confirmed to be free of mycoplasma contamination by polymerase chain reaction (PCR) (30).

### Animal studies

All mouse experiments including PDX model establishment were approved by Hokkaido University Institutional Animal Care and Use Committee (protocol number: 20–0083 and 21–0062), and conducted in accordance with the Animal Research: Reporting of *In Vivo* Experiments (ARRIVE) guidelines. Three 5-week-old female KSN/Slc mice were used for each cell line transplantation experiment. Three million HU-HSA-2 cells or one million HU-HSA-3 cells in serum-free DMEM were inoculated subcutaneously into both flanks of KSN/Slc mice anesthetized with 0.3 mg/kg medetomidine, 4 mg/kg midazolam, and 5 mg/kg butorphanol. After tumor cell inoculation, mice were recovered by intraperitoneal injection of 3 mg/kg atipamezole. Tumor volumes were calculated using the formula: volume = (length × width^2^)/2. Mice were euthanized with CO_2_ when tumors reached 1 cm^3^ in volume.

### Western blotting

SDS lysis buffer {2% SDS, 50 mM Tris–HCl (pH 6.8), and 1 mM EDTA (pH 8.0)} was added to cultured cells after washing them twice with ice-cold PBS. Whole cell lysates were then sonicated using Branson Sonifier 450 for 2 s at power setting 2. Protein concentrations were measured with TaKaRa BCA Protein Assay Kit before adding 4 × sample loading buffer {200 mM Tris–HCl buffer (pH 6.8), 8% SDS, 40% glycerol, 1% bromophenol blue, and 20% 2-mercaptoethanol} and denaturing the samples at 98 °C for 5 min. Two to five (2–5) μg of protein was separated on gradient SDS-polyacrylamide gels by electrophoresis and transferred to Immobilon-P transfer membranes. Membranes were blocked with 3% skim milk in Tris-buffered saline with 0.05% Tween 20 (TBST) for 1 h at room temperature (RT) and incubated with primary antibodies diluted in Can Get Signal Solution 1 overnight at 4 °C. Membranes were washed with TBST three times before incubating with the corresponding secondary anti-mouse or anti-rabbit IgG antibodies conjugated with horseradish peroxidase in Can Get Signal Solution 2. Signals were developed with Immobilon Western Chemiluminescent HRP substrate and visualized using an Image Quant LAS 4000 mini luminescent image analyzer. Captured data were processed using ImageJ (v1.54p) (31). Antibodies used in this study are listed in Table 1.

### Hematoxylin and eosin staining, and immunohistochemistry (IHC)

Tumor samples were obtained from canine patients presented to HUVTH with written informed consent (Table 3). Samples were fixed in 10% neutral-buffered formalin, dehydrated through an ethanol series, cleared with xylene and infiltrated with paraffin wax in Tissue-Tek VIP5 Jr. Then, the samples were embedded in paraffin wax and sliced into 2 μm-thick sections. For hematoxylin and eosin staining, tissues were deparaffinized with xylene and placed in 99, 95, 90, 80, and 70% ethanol for 2 min each in this order. After washing out the ethanol with tap water and distilled water (DW), the tissues were stained with hematoxylin for 1 min and then washed with tap water for 5 min. Then, they were stained with eosin for 1.5 min after being placed in 95% ethanol for 2 min. Remaining eosin was washed with 95% ethanol. Afterwards, the tissues were dehydrated with absolute ethanol and cleared with xylene. Finally, the tissues were mounted with Eukitt and covered with cover glasses for histopathological analysis. For IHC, after deparaffinization, the tissues were washed with PBS three times, and then antigens were retrieved in citrate buffer (pH 6.0) in a pressure cooker for 9 min. Endogenous peroxidases were inactivated with 0.3% H_2_O_2_ in methanol for 25 min at RT before blocking the tissue sections with 10% normal goat serum for single staining and 5% skim milk in PBS for double staining for 30 min at RT. For single staining, tissues were stained with anti-L-Lactyl-Histone H3 Lys18 (H3K18la) rabbit monoclonal antibody or anti-pan-lactylated lysine (Kla) rabbit monoclonal antibody overnight at 4 °C. Afterwards, the tissues were washed with PBS three times and stained with peroxidase-conjugated goat anti-rabbit IgG antibody for 30 min at RT. The slides were washed with PBS three times again, and then signals were developed by reaction with 3,3′-diaminobenzidine (DAB). For double staining, sections stained with anti-H3K18la antibody were autoclaved in citrate buffer (pH 6.0) for 2 min to deactivate the first antibody. They were washed with PBS three times and stained with anti-Iba1, CD3, and CD204 antibodies overnight at 4 °C after blocking with 5% skim milk in PBS for 30 min. Subsequently, the tissues were washed with Tris-buffered saline (TBS) three times and stained with alkaline phosphatase (AP)-conjugated goat anti-rabbit IgG or goat anti-mouse IgG antibodies for 30 min at RT. The slides were washed with TBS three times again, and then signals were developed with New Fuchsin solution. To quantify IHC results, slides were scanned with NanoZoomer 2.0-RS and analyzed with QuPath ver.0.5.1 (32). Areas within 500 μm from the tissue edge were not selected to avoid the edge effect. For intensity comparison of endothelial and HSA cells, five tumor areas per slide in each case were randomly selected. Normal endothelial cells were obtained near the tumor areas in the same section. At least a total of 1,000 tumor cells and 100 normal endothelial cells were analyzed for each case. For comparison of H3K18la and Kla intensity in HSA and endothelial cells, raw values of nucleus DAB OD mean were applied. For correlation analysis of tumor-cell H3K18la intensity and the number of immune cells, nucleus DAB OD mean values in tumor cells were normalized against those in normal endothelial cells, and the normalized values were used for quantitative analyses. Then tumor areas within each case (cases #4–13) were stratified based on H3K18la staining intensity. The thresholds for low, middle, and high H3K18la intensity were manually established for each case based on the overall staining distribution across the entire tumor area. Subsequently, five distinct regions, each larger than 1 mm^2^, were selected per case, ensuring that low, middle, and high H3K18la areas were included in the analysis. A positive staining threshold for the AP signal of each immune marker (Iba-1, CD204, CD3) was then determined manually based on visual inspection of representative positive and negative cells. Cells with a cell AP OD mean exceeding this threshold were classified as positive. QuPath was used to automatically count the number of positive cells within each area, and the results were expressed as the density of positive cells per square millimeter (cells/mm^2^).

### Cell growth assay

For cell growth assays, cells were seeded in triplicate onto 12-well plates at a density of 1.0 × 10^4^ cells per well for experiments under 10% FBS conditions, or 1.2 × 10^4^ cells per well for experiments that required FBS restriction. Cells were allowed to attach to dishes overnight in complete DMEM. On the following day (day 0), the medium was replaced with media prepared for each experimental condition, which were DMEM supplemented with or without glucose, asparagine (2 mM), and/or proline (5 mM) as indicated in each section. At each time point (0, 24, 48, 72, and 96 h), cells were washed with PBS and detached using 100 μL of 0.25% Trypsin–EDTA solution with a 5-min incubation at 37 °C. The reaction was neutralized by adding 1 mL of complete DMEM. The number of live cells was then determined using CellDrop BF with trypan blue staining.

### Plasmid construction and transfection

Single guide RNAs (sgRNAs) targeting genes of interest were designed using publicly available tools, including the CRISPR gRNA design tool from Horizon Discovery,^1^ CRISPOR^2^ (33), and CRISPR direct^3^ (34). To minimize off-target effects, sgRNAs with high specificity and efficiency scores were selected. Oligonucleotides for each selected sgRNA were synthesized, annealed, and cloned into the lentiCRISPRv2 plasmid (a gift from Feng Zhang, Addgene plasmid #52961; RRID: Addgene_52,961) (35) according to the provider’s protocol. The oligonucleotide sequences used for generating each knockout construct are listed in Table 4.

**Table 4:** sgRNA sequences.

Species	Target	sgRNA sequence
Canine	sgScr1	ATTCTCTCGACATCTGGTGG
	sgScr2	GCTCGGTAACTAACCGGTGC
	sg*SLC2A1*	AGTGTTGTAGCCAAACTGCA
	sg*ATF4*-1	TCCAGTAAAGTCCCGCGACA
	sg*ATF4*-2	TTGGTCAGTGCCTCAGACAA

Lentiviral particles were produced by transfecting lentivirus plasmids into 293 T cells. Briefly, 293 T cells were seeded in 6-well plates and grown to approximately 60–70% confluency. Cells were then co-transfected with a mixture of 4 μg of the lentiCRISPRv2-sgRNA plasmid, 0.5 μg of the packaging plasmid pCAG-HIVgp (RIKEN BRC, cat. RDB04394) (36), and 0.5 μg of the envelope plasmid pCMV-VSV-G (RIKEN BRC, cat. RDB04392) using Lipofectamine 3,000 and P3000 reagent according to the manufacturer’s protocol. pCAG-HIVgp and pCMV-VSV-G were provided by the RIKEN BRC through the National BioResource Project of the MEXT/AMED, Japan. Forty-eight hours post-transfection, the supernatant containing lentiviral particles was harvested, filtered through a 0.45 μm pore filter, and supplemented with polybrene to a final concentration of 10 μg/mL. For infection, 2 × 10^4^ HU-HSA-2 or HU-HSA-3 cells were incubated with the virus-containing supernatant for 8 h. Following infection, the viral medium was replaced with complete DMEM, and the cells were cultured for 72 h. Subsequently, cells stably expressing Cas9 and sgRNAs were selected by culture in complete DMEM containing puromycin at a concentration of 2 μg/mL for HU-HSA-2 or 4 μg/mL for HU-HSA-3.

### Extracellular flux analysis

Extracellular flux experiments were performed according to the manufacturer’s protocol. Briefly, 2.5 × 10^3^ HU-HSA-2 or 2.0 × 10^3^ HU-HSA-3 cells were seeded on assay plates coated with 0.1% gelatin and incubated overnight. On the next day, the medium was replaced with complete DMEM with or without glucose. After 48 h incubation, the medium was replaced with FBS-free DMEM for flux analysis, which contained 4 mM L-glutamine alone or with 25 mM D(+)-glucose. Cells were incubated for at least 1 h at 37 °C without CO_2_ regulation. Assay solutions were loaded on each assay cartridge well. For the ATP rate assay, 1.5 μM oligomycin and 0.5 μM rotenone/antimycin A were applied. For mitochondrial oxidation assay, 3 μM BPTES, 2 μM UK5099 and 4 μM etomoxir were applied. Oxygen consumption rate and extracellular acidification rate were measured using a Seahorse XFp Analyzer. For normalization, cells were stained using Hoechst 33342 after measurement, and the well images were acquired using EVOS FL at 10 × magnification. For each well, three representative fields were randomly selected for analysis. The number of cells was quantified using ImageJ software (National Institutes of Health, United States). Briefly, images were duplicated and converted to 8-bit grayscale. A binary mask was created using the Default auto-threshold algorithm with the background set to black. To separate touching or overlapping nuclei, the Watershed command was applied. Finally, particles were analyzed using the Analyze Particles function, counting objects with a size range of 50–500 square pixels. The average number of cells in each area was used for normalizing oxygen consumption rate or extracellular acidification rate values.

### Cleavage under targets and tagmentation CUT&Tag

CUT&Tag experiments were conducted using a CUT&Tag Assay Kit following the manufacturer’s protocol with slight modifications. Briefly, 2.5 × 10^5^ HU-HSA-2 or HU-HSA-3 cells were prepared for each reaction (in duplicate for pan-Kla samples and singly for H3K4me3, H3K27ac, and RNAPII-Ser5P samples). Cells were washed with 1.0 mL Complete Wash Buffer twice and incubated with activated beads for 5 min at RT, and then antibodies (pan-Kla, H3K4me3, H3K27ac, and RNAPII-Ser5P) were added and incubated for 1.5 h at RT. Afterwards, cells were incubated with the secondary antibody (Goat Anti-Rabbit IgG [H + L], 1:50) for 30 min at RT. pAG-Tn5 was introduced to the cells and incubated for 1 h at RT followed by washing with 500 μL Digitonin Buffer twice. Tagmentation was initiated by adding magnesium chloride and incubating for 1 h at 37 °C followed by washing with 500 μL High Salt Digitonin Buffer twice. To stop tagmentation, 6.75 μL 0.5 M EDTA, 8.25 μL 10% SDS, and 1.5 μL Proteinase K were added to the reaction, and DNA was then purified. For DNA amplification, PCR was performed on purified DNA fragments with index primers using PCR Master Mix. The process consists of 72 °C for 5 min, 13 cycles of 98 °C for 40 s and 63 °C for 10 s, and 72 °C for 1 min. Amplified DNA was purified with AMpure XP. DNA concentration was measured with Qubit using a dsDNA High Sensitivity kit. DNA quality was checked with TapeStation using High Sensitivity D5000 ScreenTape. DNA libraries were submitted to Rhelixa Co., Ltd. (Tokyo, Japan) and sequenced with the Illumina NovaSeq X Plus (Illumina, CA, United States) to generate a minimum of 20 million paired-end 150 bp reads for pan-Kla samples cultured under 0 mM glucose condition and 13.3 million paired-end 150 bp reads for pan-Kla cultured under 25 mM glucose condition and for H3K4me3, H3K27ac, and RNAPII-Ser5P samples cultured with/without glucose. Sequencing reads were qualified with fastp v0.26.0 (37) and mapped to the CanFam3.1 canine reference genome from Ensembl using Bowtie2 v2.5.4 (38). For sample normalization, the sum-of-fragments coverage was determined by calculating the number of fragments with lengths of 1 to 1,000 bp from bedpe files converted from mapped BAM files using bedtools v2.31.1 (39). Raw bedGraph files converted from BAM files using bamCoverage (deepTools v3.5.6) (40) were normalized by the sum-of-fragments coverage and then visualized using Integrative Genome Viewer (IGV). For visualizing transcription start sites (TSSs), computeMatrix (deepTools) in reference-point mode (default settings) was used, and the resulting matrix was visualized by plotProfile. For visualizing gene-body heatmaps and genomic annotation, plotPeakProf and plotAnnoPie in ChIPseeker v3.21 were used, respectively (41, 42). To identify significantly enriched peaks in the 0 mM glucose condition relative to the 25 mM condition, peak calling was performed using MACS2 (v2.2.9.1) (43). The 0 mM glucose samples were designated as the treatment (−t), and the 25 mM glucose samples were used as the control (−c). The false discovery rate (FDR) threshold (−q) was set to 0.1 for pan-Kla and H3K4me3 samples, and 0.001 for RNAPII-Ser5P samples. Afterwards, significant peaks were then annotated to genomic features using HOMER (v5.1) (44) with a custom annotation file generated from the CanFam3.1 canine reference genome (Ensembl). Gene ontology analysis was conducted using PANTHER Pathways in PANTHER v18.0 (45, 46).

### mRNA-sequencing (mRNA-seq)

HU-HSA-2 and HU-HSA-3 were cultured under 0 mM or 25 mM glucose conditions for 48 h in triplicate. Total RNA was extracted using a NucleoSpin RNA isolation kit according to the manufacturer’s instructions. RNA samples were submitted to Rhelixa Co., Ltd. for further analyses. mRNA-seq libraries were constructed using a NEBNext Ultra II Directional RNA Library Prep Kit and sequenced with the Illumina NovaSeq X Plus platform to generate a minimum of 40 million paired-end 150-bp reads. Sequencing reads were mapped to the CanFam3.1 canine reference genome using STAR, and expression levels were estimated using RSEM (47, 48). Differential expression was analyzed with edgeR 3.42.0, and pathway enrichment was evaluated with gene set enrichment analysis (GSEA) v4.4.0 (49–51). To compare transcriptional profiles of our HSA cell lines with other canine cells, we analyzed our mRNA-seq data of HSA cell lines and 29 publicly available canine RNA-seq datasets (BioProject accessions PRJNA719562, PRJNA803064, PRJNA590267, PRJNA689618, and PRJNA786902) (Table 5). All FASTQ files were aligned to the CanFam4 reference genome with STAR, and gene expression levels were estimated with RSEM. Read counts were analyzed in R v4.3.2. Genes with low counts per million (CPM) were filtered with edgeR (filterByExpr), library sizes were normalized by the trimmed mean of M-values (TMM), and precision weights were estimated with limma-voom. Principal-component analysis was performed on log_2_CPM values after removing batch effects using limma 3.56.2. Mean ± SD expression of endothelial markers was summarized per biological group and visualized with ggplot2.

**Table 5:** Summary of canine cell sequencing datasets.

Experiment accession	Experiment title	Organism name	Instrument	Study accession	Sample accession	Total size, Mb	Total spots	Total bases
SRX10509643	GSM5225596: endothelial cells coronary artery dog A; *Canis lupus familiaris*; RNA-Seq	*Canis lupus familiaris*	NextSeq 500	SRP313354	SRS8633736	158	11425142	863734867
SRX10509644	GSM5225597: endothelial cells coronary artery dog C; *Canis lupus familiaris*; RNA-Seq	*Canis lupus familiaris*	NextSeq 500	SRP313354	SRS8633737	167	11893399	899031286
SRX10509645	GSM5225598: endothelial cells femoral artery dog A; *Canis lupus familiaris*; RNA-Seq	*Canis lupus familiaris*	NextSeq 500	SRP313354	SRS8633738	160	11500946	869197148
SRX10509648	GSM5225601: endothelial cells pulmonary artery dog A; *Canis lupus familiaris*; RNA-Seq	*Canis lupus familiaris*	NextSeq 500	SRP313354	SRS8633741	170	11976954	905294855
SRX10509649	GSM5225602: endothelial cells pulmonary artery dog B; *Canis lupus familiaris*; RNA-Seq	*Canis lupus familiaris*	NextSeq 500	SRP313354	SRS8633742	178	12770805	965107572
SRX10509650	GSM5225603: endothelial cells pulmonary artery dog C; *Canis lupus familiaris*; RNA-Seq	*Canis lupus familiaris*	NextSeq 500	SRP313354	SRS8633743	168	12224963	924067882
SRX9779362	GSM5005184: D17 parental rep 1; *Canis lupus familiaris*; RNA-Seq	*Canis lupus familiaris*	Illumina HiSeq 2,500	SRP300293	SRS7966796	2,343	44634511	5579313875
SRX9779363	GSM5005185: D17 parental rep 2; *Canis lupus familiaris*; RNA-Seq	*Canis lupus familiaris*	Illumina HiSeq 2,500	SRP300293	SRS7966797	2,175	41414982	5176872750
SRX9779364	GSM5005186: D17 parental rep 3; *Canis lupus familiaris*; RNA-Seq	*Canis lupus familiaris*	Illumina HiSeq 2,500	SRP300293	SRS7966798	2,255	43027350	5378418750
SRX9779368	GSM5005190: HMPOS parental rep 1; *Canis lupus familiaris*; RNA-Seq	*Canis lupus familiaris*	Illumina HiSeq 2,500	SRP300293	SRS7966802	1,501	26810051	3351256375
SRX9779369	GSM5005191: HMPOS parental rep 2; *Canis lupus familiaris*; RNA-Seq	*Canis lupus familiaris*	Illumina HiSeq 2,500	SRP300293	SRS7966803	1,649	29447128	3680891000
SRX9779370	GSM5005192: HMPOS parental rep 3; *Canis lupus familiaris*; RNA-Seq	*Canis lupus familiaris*	Illumina HiSeq 2,500	SRP300293	SRS7966804	1896	33553175	4194146875
SRX13340456	GSM5720812: 1508-1_S1_L001; *Canis lupus familiaris*; RNA-Seq	*Canis lupus familiaris*	NextSeq 500	SRP349649	SRS11258887	204	7055040	532748267
SRX13340457	GSM5720813: 1508-1_S1_L002; *Canis lupus familiaris*; RNA-Seq	*Canis lupus familiaris*	NextSeq 500	SRP349649	SRS11258886	210	7220453	545263057
SRX13340458	GSM5720814: 1508-1_S1_L003; *Canis lupus familiaris*; RNA-Seq	*Canis lupus familiaris*	NextSeq 500	SRP349649	SRS11258888	212	7244256	547052616
SRX13340459	GSM5720815: 1508-1_S1_L004; *Canis lupus familiaris*; RNA-Seq	*Canis lupus familiaris*	NextSeq 500	SRP349649	SRS11258889	210	7129627	538409294
SRX13340460	GSM5720816: 1508-2_S7_L001; *Canis lupus familiaris*; RNA-Seq	*Canis lupus familiaris*	NextSeq 500	SRP349649	SRS11258891	185	6400694	483231680
SRX13340461	GSM5720817: 1508-2_S7_L002; *Canis lupus familiaris*; RNA-Seq	*Canis lupus familiaris*	NextSeq 500	SRP349649	SRS11258890	190	6549323	494498009
SRX13340462	GSM5720818: 1508-2_S7_L003; *Canis lupus familiaris*; RNA-Seq	*Canis lupus familiaris*	NextSeq 500	SRP349649	SRS11258893	193	6575715	496464875
SRX13340463	GSM5720819: 1508-2_S7_L004; *Canis lupus familiaris*; RNA-Seq	*Canis lupus familiaris*	NextSeq 500	SRP349649	SRS11258892	191	6469921	488494527
SRX13340464	GSM5720820: 1508-3_S8_L001; *Canis lupus familiaris*; RNA-Seq	*Canis lupus familiaris*	NextSeq 500	SRP349649	SRS11258895	186	6402997	483476466
SRX13340465	GSM5720821: 1508-3_S8_L002; *Canis lupus familiaris*; RNA-Seq	*Canis lupus familiaris*	NextSeq 500	SRP349649	SRS11258894	191	6531844	493239108
SRX13340466	GSM5720822: 1508-3_S8_L003; *Canis lupus familiaris*; RNA-Seq	*Canis lupus familiaris*	NextSeq 500	SRP349649	SRS11258898	194	6570878	496171220
SRX13340467	GSM5720823: 1508-3_S8_L004; *Canis lupus familiaris*; RNA-Seq	*Canis lupus familiaris*	NextSeq 500	SRP349649	SRS11258896	191	6432303	485718048
SRX13340420	GSM5720800: 1506-1_S3_L001; *Canis lupus familiaris*; RNA-Seq	*Canis lupus familiaris*	NextSeq 500	SRP349649	SRS11245299	179	6061797	457666042
SRX13340421	GSM5720801: 1506-1_S3_L002; *Canis lupus familiaris*; RNA-Seq	*Canis lupus familiaris*	NextSeq 500	SRP349649	SRS11245300	186	6308546	476327052
SRX13340422	GSM5720802: 1506-1_S3_L003; *Canis lupus familiaris*; RNA-Seq	*Canis lupus familiaris*	NextSeq 500	SRP349649	SRS11245301	186	6220877	469693213
SRX13340423	GSM5720803: 1506-1_S3_L004; *Canis lupus familiaris*; RNA-Seq	*Canis lupus familiaris*	NextSeq 500	SRP349649	SRS11245302	186	6148315	464223596
SRX13340448	GSM5720804: 1506-2_S11_L001; *Canis lupus familiaris*; RNA-Seq	*Canis lupus familiaris*	NextSeq 500	SRP349649	SRS11245307	178	6012595	454001258
SRX13340449	GSM5720805: 1506-2_S11_L002; *Canis lupus familiaris*; RNA-Seq	*Canis lupus familiaris*	NextSeq 500	SRP349649	SRS11245308	179	6066321	458074581
SRX13340450	GSM5720806: 1506-2_S11_L003; *Canis lupus familiaris*; RNA-Seq	*Canis lupus familiaris*	NextSeq 500	SRP349649	SRS11245309	185	6165693	465573259
SRX13340451	GSM5720807: 1506-2_S11_L004; *Canis lupus familiaris*; RNA-Seq	*Canis lupus familiaris*	NextSeq 500	SRP349649	SRS11245310	180	5930455	447816603
SRX13340452	GSM5720808: 1506-3_S6_L001; *Canis lupus familiaris*; RNA-Seq	*Canis lupus familiaris*	NextSeq 500	SRP349649	SRS11245311	192	6654762	502477497
SRX13340453	GSM5720809: 1506-3_S6_L002; *Canis lupus familiaris*; RNA-Seq	*Canis lupus familiaris*	NextSeq 500	SRP349649	SRS11245312	197	6806659	513974551
SRX13340454	GSM5720810: 1506-3_S6_L003; *Canis lupus familiaris*; RNA-Seq	*Canis lupus familiaris*	NextSeq 500	SRP349649	SRS11245313	200	6838721	516388344
SRX13340455	GSM5720811: 1506-3_S6_L004; *Canis lupus familiaris*; RNA-Seq	*Canis lupus familiaris*	NextSeq 500	SRP349649	SRS11245314	198	6729910	508183809
SRX7177537	GSM4175965: UUA—uninfected cells from uninfected well; *Canis lupus familiaris*; RNA-Seq	*Canis lupus familiaris*	Illumina NovaSeq 6,000	SRP230456	SRS5684513	1,515	17108657	5132597100
SRX7177538	GSM4175966: UUB—uninfected cells from uninfected well; *Canis lupus familiaris*; RNA-Seq	*Canis lupus familiaris*	Illumina NovaSeq 6,000	SRP230456	SRS5684514	1,424	16006488	4801946400
SRX7177539	GSM4175967: UUC—uninfected cells from uninfected well; *Canis lupus familiaris*; RNA-Seq	*Canis lupus familiaris*	Illumina NovaSeq 6,000	SRP230456	SRS5684515	1,537	17429567	5228870100
SRX7177540	GSM4175968: UUD—uninfected cells from uninfected well; *Canis lupus familiaris*; RNA-Seq	*Canis lupus familiaris*	Illumina NovaSeq 6,000	SRP230456	SRS5684516	1742	19444560	5833368000
SRX14030411	RNA-Seq of *Canis lupus familiaris*: Fibroblast	*Canis lupus familiaris*	Illumina MiSeq	SRP358051	SRS11866026	413	14991035	749551750
SRX14030410	RNA-Seq of *Canis lupus familiaris*: Fibroblast	*Canis lupus familiaris*	Illumina MiSeq	SRP358051	SRS11866024	996	36050619	1802530950
SRX14030407	RNA-Seq of *Canis lupus familiaris*: Fibroblast	*Canis lupus familiaris*	Illumina MiSeq	SRP358051	SRS11866022	495	17742386	887119300
SRX14030406	RNA-Seq of *Canis lupus familiaris*: Fibroblast	*Canis lupus familiaris*	Illumina MiSeq	SRP358051	SRS11866021	1,313	47701815	2385090750

### RT-qPCR

Total RNA was extracted with TriPure Isolation Reagent according to the manufacturer’s instructions. Reverse transcription was performed using PrimeScript II 1st Strand cDNA Synthesis Kit for 1 μg of total RNA per sample according to the manufacturer’s instructions. qPCR samples were prepared using KAPA SYBR FAST qPCR Kit Master Mix (2×) ABI Prism. The reaction solution contained 1 × KAPA SYBR FAST qPCR Master Mix, 200 nM forward and reverse primers, 1 μL cDNA, and UltraPure DNase/RNase-free distilled water (UPDW). The samples were applied in triplicate and analyzed on a StepOne Real-Time PCR System. Samples were denatured at 95 °C for 20 s followed by 40 cycles of 95 °C for 3 s and 60 °C for 30 s. RT-qPCR was performed using the primers listed in Table 6. Results were normalized using the geometric mean of reference genes (*RPL32*, *ACTB*, *B2M*, *HMBS*, *TBP*, and *YWHAZ*), which were selected from potential internal controls by geNorm (52). No-template controls (UPDW) and no-RT samples were used as negative controls. No amplification was detected for any primer set. Gene sequences were obtained from Ensembl. Primer3 ver 3.3.0 was used to design primers targeting 80–150 bp products. The primers were designed to bind all splice variants and to span exon-exon junctions where possible. NCBI BLAST was used to confirm that each primer set did not detect other genes. Primer-set specificity was evaluated by verifying a single peak in the melt curve. Relative expression levels were calculated by setting the 0 h sample to 1.

**Table 6:** qPCR primer sequences.

Species	Target	Sequence (forward)	Sequence (reverse)	Gene ID
Canine	*RPL32*	TGGTTACAGGAGCAACAAGAAA	GCACATCAGCAGCACTTCA	ENSCAFG00000004821
	*TBP*	ATAAGAGAGCCCCGAACCAC	TTCACATCACAGCTCCCCAC	ENSCAFG00000004119
	*YWHAZ*	CGAAGTTGCTGCTGGTGA	TTGCATTTCCTTTTTGCTGA	ENSCAFG00000000580
	*ACTB*	CCAGCAAGGATGAAGATCAAG	TCTGCTGGAAGGTGGACAG	ENSCAFG00000016020
	*HMBS*	TCACCATCGGAGCCATCT	GTTCCCACCACGCTCTTCT	ENSCAFG00000012342
	*B2M*	ACGGAAAGGAGATGAAAGCA	CCTGCTCATTGGGAGTGAA	ENSCAFG00000013633
	*PECAM1*	AACTTCACCATCCAGAAGG	TCCACTGGGGCTATCACC	ENSCAFG00000011740
	*VWF*	AAGCAGACGATGGTGGATTC	AATGTCCAGGAATGGCTCAG	ENSCAFG00000015228
	*KDR*	GGTATGGTCCTTGCCTCAGA	CAGTGGTATCCGTGTCATCG	ENSCAFG00000002079
	*ATF4*	CTTAAGCCATGGCGCTTTTC	GGAATGTGCTTAATTCGAAGGTG	ENSCAFG00000001324
	*ASNS*	TTGGGTTTTGTGCCACCATG	AGAAAGGAAGAGGGGAAAGCTG	ENSCAFG00000002222
	*SLC7A11*	ATTCATGTCCGCAAGCACAC	TGCCAGCCCAATAAAAAGCC	ENSCAFG00000003749
	*SESN2*	TTAGCTGCTTTTGGCGTCTG	TGCAGAAACTCAGCCATGTG	ENSCAFG00000011842
	*CHAC1*	AGATCATGAGGGCTGCACTTG	TAGCCGCCAAGTACTGCTTC	ENSCAFG00000009414
	*DDIT3*	GCGGATCATGTTGAAGATGAGC	TCAGCTGCCATCTCTACAGTTG	ENSCAFG00000030112
	*MTHFD2*	TGTAGATGGCCTCCTTGTTCAG	AACAGCGTTGCAGACCTTTC	ENSCAFG00000008716
	*PYCR1*	GCCACACATCATCCCCTTTATC	AACGCCATCAGCTTCTTCTC	ENSCAFG00000005906
	*PYCR2*	TGTCGGCTCACAAGATCATAGC	TCACCGTCTCCTTGTTGTTCC	ENSCAFG00000016140
	*ALDH18A1*	ATGGAAGCCAAGGTGAAAGC	TGGGTTCCGTTGGCAATAAC	ENSCAFG00000008339

### Single-cell transcriptome analysis

Tumor tissues were harvested from two PDX models under anesthesia as described in the Animal Study section. Tumor tissues were washed with PBS to remove excess blood and trimmed to carefully remove mouse-derived adipose and connective tissues. The remaining tumor tissue was mechanically dissociated by mincing it into small fragments (~1–2 mm cubes) using sterile scalpels. Tissue fragments were then washed with PBS containing 0.1% bovine serum albumin (BSA) to prevent cell aggregation, and then subjected to two rounds of RBC lysis using an NH₄Cl-based buffer with gentle agitation for 5 min. Following a wash with PBS/BSA, the tissue fragments were enzymatically digested in a solution containing 3 mg/mL collagenase I and 1 μg/mL DNase I in DMEM for 50 min at 37 °C with intermittent mixing. The digested tissue was gently homogenized by passing through 18G and 23G needles. The resulting cell suspension was then passed through a 40 μm cell strainer to remove any remaining clumps. After another wash and RBC lysis step to ensure purity, dead cells were depleted using the Dead Cell Removal Kit according to the manufacturer’s protocol. The number of viable cells was determined with trypan blue staining. The cell concentration was adjusted to approximately 1,000 cells/μL. Five thousand cells per sample were loaded onto a Chromium Controller for single-cell capture. Single-cell gene expression libraries were prepared using the Chromium Next GEM Single Cell 3’ Reagent Kits v3.1 for Dual Index following the manufacturer’s protocol. The quality and fragment size distribution of the final libraries were assessed using an Agilent Bioanalyzer 2,100 with a High Sensitivity DNA Kit. Libraries were then pooled and sequenced on an Illumina NovaSeq 6,000 platform with a targeted sequencing depth of 800 million reads per sample.

For data analysis, canine-specific reads were first extracted using XenoCell with default settings (53), and mapping and alignment were conducted by Cell Ranger (10X Genomics). Data were processed with Seurat ver. 4.2.0 using R ver. 4.5.0 in Rstudio ver. 2025.05.0. Metascape analysis for cluster 10 was conducted using Metascape (54).

### Spatial transcriptome analysis

Patient information is detailed in Table 3. A tumor tissue sample obtained from a splenectomy was embedded in Optimal Cutting Temperature (OCT) compound, snap-frozen, and stored at −80 °C until use. The frozen tissue was sectioned at 10 μm thickness and placed onto a Stereo-seq chip. Stereo-seq library preparation, sequencing, and primary data analysis were performed by AZENTA (Chelmsford, MA, United States). Libraries were sequenced on a DNBSEQ platform, generating 1.0–1.5 G of paired-end reads, ensuring that >75% of reads achieved a Phred score ≥ Q30. Raw sequencing reads were demultiplexed using the DNBSEQ platform’s built-in software, and the quality of the raw data was assessed using fastp v.0.20.0. The final data processing, including alignment to the canine reference genome (ROS_Cfam_1.0) and spatial expression mapping, was performed using the Stereo-seq Analysis Workflow (SAW) pipeline.

### Migration assay

HU-HSA-2 and HU-HSA-3 were cultured under 0 mM or 25 mM glucose conditions for 24 h in 6-well plates. Then, the cells were co-cultured with RAW264 cells seeded on ThinCert Cell-culture inserts for 24 h. RAW264 cells on the upper surface of the inserts were removed with a cotton swab. Migrated RAW264 cells on the lower membrane surface were fixed with 4% paraformaldehyde for 30 min at RT, and then stained with 0.01% crystal violet for 30 min at RT. The number of cells was counted manually in ten fields at 200 × under a light microscope (BX-41).

### Conditioned medium assay

HU-HSA-3 cells were cultured in DMEM with or without 25 mM glucose for 48 h. The supernatant was collected as conditioned medium and used for further analysis after filtering through a 0.2 μm pore filter. D(+)-glucose was added to the conditioned medium obtained from the 0 mM glucose condition to a final concentration of 5.6 mM to allow RAW264 cells to survive. RAW264 cells were cultured with the conditioned medium (glucose 5.6 mM or 25 mM) or complete DMEM (glucose 5.6 mM or 25 mM) for 24 h. Afterward, total RNA was harvested for RT-qPCR.

### Metabolome analysis

1.0 × 10^5^ HU-HSA-3 cells were seeded in 10 cm dishes with regular DMEM cell culture medium in triplicate and incubated overnight. The next day, the medium was changed to DMEM without glucose and glutamine (#042-32255, Fujifilm Wako, custom order lacking glutamine) supplemented with 4 mM ^13^C_5_ L-glutamine. Cells were harvested at 0, 0.5, 3, 24, and 48 h after changing the medium. Briefly, cells were washed with 3.4% erythritol twice after aspirating cell culture medium. 800 μL methanol and 10 μM internal standard were added, and then the extracted solution was centrifuged at 2,300 × g, 4 °C for 5 min. Supernatant was collected and ultrafiltered with a 5 kDa cutoff filter at 9,100 × g, 4 °C for 3 h to remove proteins. Samples were then submitted to Human Metabolome Technology (Yamagata, Japan) for further analyses. Metabolic products were analyzed by an Agilent CE-TOF MS system (Agilent Technologies) (55) with a fused silica capillary (i.d. 50 μm × 80 cm in total length) in cation and anion modes. The signal-to-noise ratio for each peak was calculated, and peaks with a ratio more than 3 were used for the analysis. Using mass-to-charge ratios (m/z) and migration time of each peak, metabolic products were determined based on a metabolic product library from Human Metabolome Technology. For quantification of metabolic products, the concentration of total isotope ions for each product was calculated and normalized with the internal standard (56, 57).

### Cell viability assay

Two thousand cells were seeded in 96-well cell culture plates and cultured in 100 μL DMEM corresponding to each experimental condition. On the next day, cells were treated with either dimethyl sulfoxide (DMSO), tunicamycin, or salubrinal each at five different concentrations (10 μg/mL, 1 μg/mL, 0.1 μg/mL, 0.01 μg/mL, and 0.001 μg/mL for tunicamycin; 100 μM, 10 μM, 1 μM, 0.1 μM, and 0.01 μM for salubrinal). Survival rates were analyzed using Cell Counting Kit-8 (CCK-8) according to the manufacturer’s instructions with slight modifications. Briefly, 10 μL of CCK-8 solution was added to each well 48 h after adding DMSO or either inhibitor. After a 2-h incubation, 10 μL of 0.1% SDS solution was added to stop the reaction and the absorbance at 450 nm was measured with a microplate reader MTP-320. Survival rates were calculated by setting the absorbance of DMSO-treated samples as 100%. KyPlot v5.0 software (KyensLab, Inc., Tokyo, Japan) was used to draw survival curves (58).

### Statistical analysis

All statistical analyses were performed using R software (version 4.5.0; R Foundation for Statistical Computing, Vienna, Austria). A *p* value of less than 0.05 was considered statistically significant. Data distribution was first assessed for normality using the Shapiro–Wilk test. For comparisons between two independent groups, the two-tailed Student’s *t* test was used for normally distributed data. For comparisons among more than two groups, one-way analysis of variance (ANOVA) was performed, followed by Dunnett’s *post hoc* test to compare each experimental group against the control group. For experiments involving two independent variables, two-way ANOVA was used, followed by an appropriate post hoc test for specific comparisons. *In vivo* tumor growth curves were analyzed using two-way ANOVA with repeated measures, and differences at the final time point were assessed using Dunnett’s multiple comparisons test. Correlations between H3K18la intensity and immune cell density were assessed using Pearson’s correlation coefficient.

### AI-assisted tools

We used OpenAI ChatGPT (model o3, and 5.2 accessed June 2025–December 2025) and Google Gemini 2.5 Pro (accessed September 2024–March 2025) for English proofreading and for methodological suggestions for bioinformatic analyses. Representative prompts were “Please check the grammar of the attached document. Results should be shown as a list with line numbers and correct grammar” for English proofreading, and “Please explain common methods for creating peak plots of CUT&Tag signals around the TSS regions” for methodological suggestions. All outputs were reviewed and edited by the authors. No AI-generated data were used.

## Results

### Establishment of canine hemangiosarcoma cell lines and PDX models

We established two canine HSA cell lines from splenic tumors of two dog patients (HU-HSA-2 and HU-HSA-3, Supplementary Figure S1A; patient details in Table 3). They expressed endothelial-marker genes and proteins (CD31, vWF, and KDR) (Supplementary Figures S1B–D). Gene expression profiles, however, were more similar to those of fibroblasts than to those of normal femoral and pulmonary arterial endothelial cells, which might reflect undifferentiated features of neoplastic endothelial cells (Supplementary Figure S1E; Table 5). When transplanted subcutaneously into nude mice, both cell lines developed tumors that recapitulated morphological features of HSA such as blood-filled capillaries and proliferation of either spindle (HU-HSA-2) or round to oval (HU-HSA-3) tumor cells (Supplementary Figures S1F,G). HU-HSA-2 cells develop tumors at four of six inoculation sites, and tumor growth rates were variable across the sites where tumors formed, whereas HU-HSA-3 formed tumors at all inoculation sites and showed consistent tumor growth (Supplementary Figure S1F). PDX models were also generated from three dog patients (HU-HSA-2, HU-HSA-3, and HU-HSA-1). They retained histological features of the corresponding patient tumors and expressed endothelial markers (CD31 and vWF) (Supplementary Figure S1G). Short tandem repeat analysis with a commercial canine genotyping kit confirmed the canine origin and the unique identity of each cell line and PDX model (Supplementary Figure S1H). We used these resources in subsequent experiments.

### Glucose is the major source of histone lactylation in HSA cells

**Figure 1:**
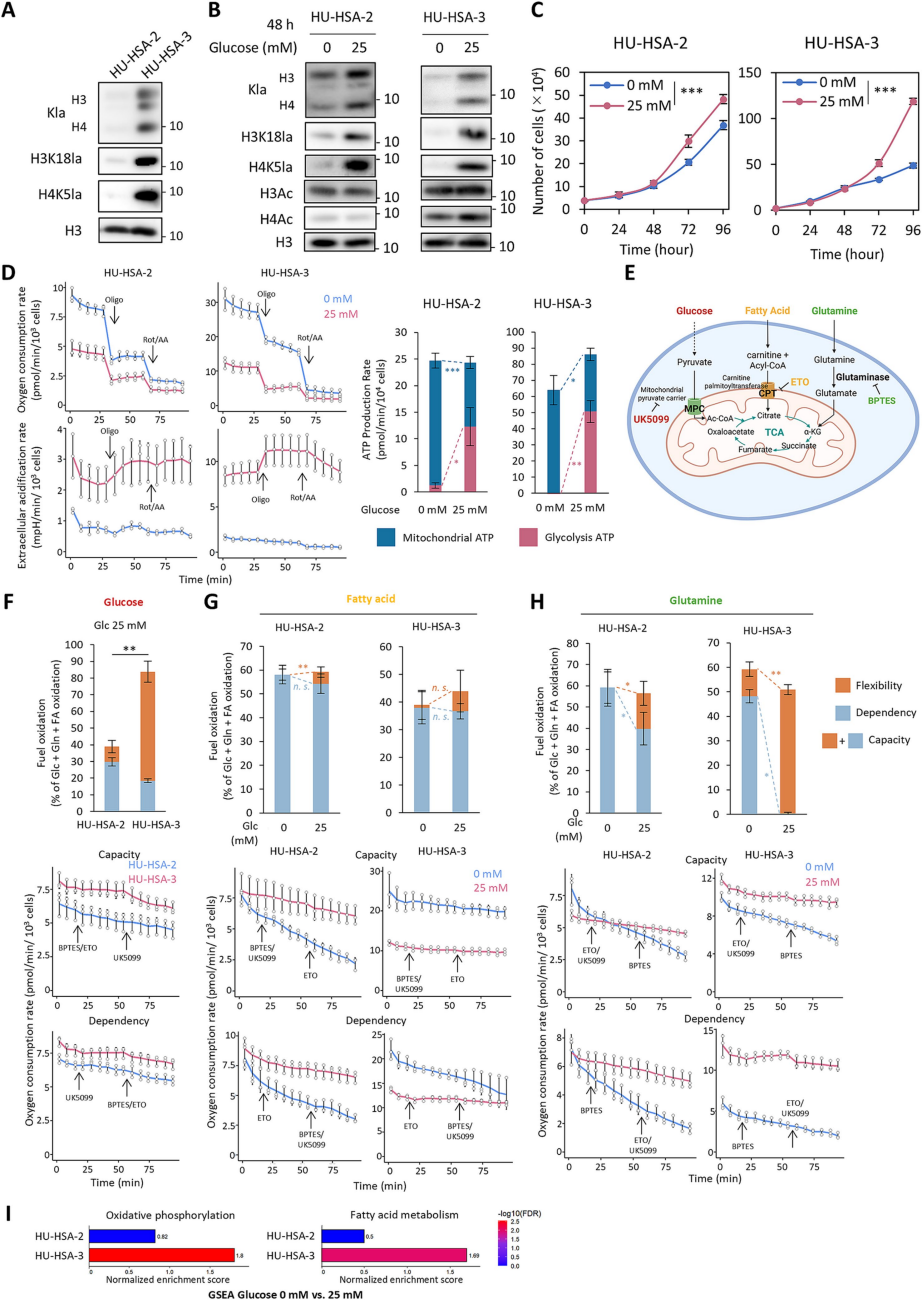
Glucose starvation reduces global histone lactylation levels and reprograms HSA cell metabolism. **(A)** Western blot analysis of histone lactylation and total histone H3 levels in HSA cell lines under 25 mM glucose condition. **(B)** Western blot analysis of histone lactylation and acetylation, and total histone H3 levels in HSA cell lines. **(C)** Growth curves of HSA cell lines cultured with or without glucose over 96 h *in vitro*. **(D)** Extracellular flux analysis of HSA cell lines. (Left) Oxygen consumption rate (OCR) and extracellular acidification rate (ECAR) traces. (Right) Calculated ATP production rates from mitochondria and glycolysis. **(E)** A schematic diagram of the seahorse Mito Fuel Flex Test. Created with BioRender. **(F–H)** Mitochondrial fuel oxidation analysis in HSA cell lines. (Top) Calculated fuel oxidation, dependency, and flexibility for glucose **(F)**, fatty acids **(G)**, and glutamine **(H)**. (Bottom) OCR traces during sequential inhibition of fuel pathways. **(I)** GSEA from mRNA-seq in HSA cell lines. HSA cells were cultured for 48 h with or without glucose in **(B,C,F–I)**. Data are presented as mean ± SD (*n* = 3). *n.s*., Not significant. *p < 0.05. **p < 0.01. ***p < 0.001. Two-way ANOVA for (C). Student’s *t* test for extracellular flux analyses.

To investigate the role of histone lactylation, we first evaluated global histone lactylation levels in the newly established HSA cell lines under regular or nutrient-deficient conditions. Under regular culture condition (25 mM glucose, 4 mM glutamine), HU-HSA-3 cells exhibited markedly stronger signals than HU-HSA-2 cells (Figure 1A). Glucose deprivation (0 mM glucose, 4 mM glutamine) for 48 h significantly decreased global pan-H3 and H4 lactylation, H3K18la, and H4K5la levels in both cell lines without altering global histone acetylation levels (H3Ac, H4Ac) (Figure 1B) and resulted in modest cell growth retardation over 96 h (Figure 1C). Polyclonal knockout of *SLC2A1* (GLUT1), a glucose transporter, by the CRISPR/Cas9 system also exhibited a significant reduction in global histone lactylation levels (Supplementary Figure S2A), although no growth inhibition was observed *in vitro* and *in vivo* (Supplementary Figures S2B,C). This could be explained by metabolic adaptation acquired during the prolonged establishment period of the knockout cells. In contrast to glucose, glutamine deprivation (25 mM glucose, 0 mM glutamine) did not affect global histone lactylation levels (Supplementary Figure S2D). These results suggest that glucose is the major source of histone lactylation in canine HSA cell lines.

Next, we assessed the metabolic status of HSA cell lines and the effects of glucose deprivation by extracellular flux analysis. Although our HSA cell lines retained endothelial characteristics, they relied comparably on glycolysis and OXPHOS for ATP production (Figure 1D). Glucose deprivation for 48 h induced a near-complete metabolic shift toward OXPHOS, while overall ATP production rates remained comparable or slightly decreased (Figure 1D). To further evaluate nutrient dependency, flexibility, and capacity, we sequentially inhibited major mitochondrial fuel pathways using UK5099 for mitochondrial pyruvate carrier, BPTES for glutaminase, and etomoxir (ETO) for carnitine palmitoyltransferase 1 (Figure 1E). HU-HSA-3 cells consumed more glucose than HU-HSA-2 (Figure 1F), which may explain why the higher global histone lactylation levels were observed in HU-HSA-3 (Figure 1A). Upon glucose deprivation for 48 h, both cell lines increased their reliance on alternative mitochondrial fuels. Changes in fatty acid oxidation parameters were modest and differed between cell lines (Figure 1G). No statistically significant change was observed in HU-HSA-3, whereas HU-HSA-2 showed only a small, albeit statistically significant, change. Glutamine oxidation dependency increased in both cell lines. A more robust change was observed in HU-HSA-3 than in HU-HSA-2 (Figure 1H). Consistent with these functional data, GSEA of mRNA-seq data showed that HU-HSA-3 cells under glucose deprivation for 48 h displayed enrichment of OXPHOS and fatty acid metabolism/oxidation pathways, whereas comparable enrichment was not observed in HU-HSA-2 cells (Figure 1I). This cell line–dependent transcriptional response is consistent with the more pronounced alteration in nutrient dependency observed in HU-HSA-3.

Taken together, our results demonstrate that glucose is the major source of histone lactylation in HSA cell lines and that glucose restriction shifts cellular metabolism toward mitochondrial respiration. This shift is accompanied by cell line-dependent changes in reliance on non-glucose mitochondrial substrates (particularly glutamine oxidation) and by transcriptional activation of OXPHOS and fatty acid metabolism pathways in HU-HSA-3.

### Lysine lactylation is enriched at TSSs and modulates gene expression during glucose starvation

**Figure 2:**
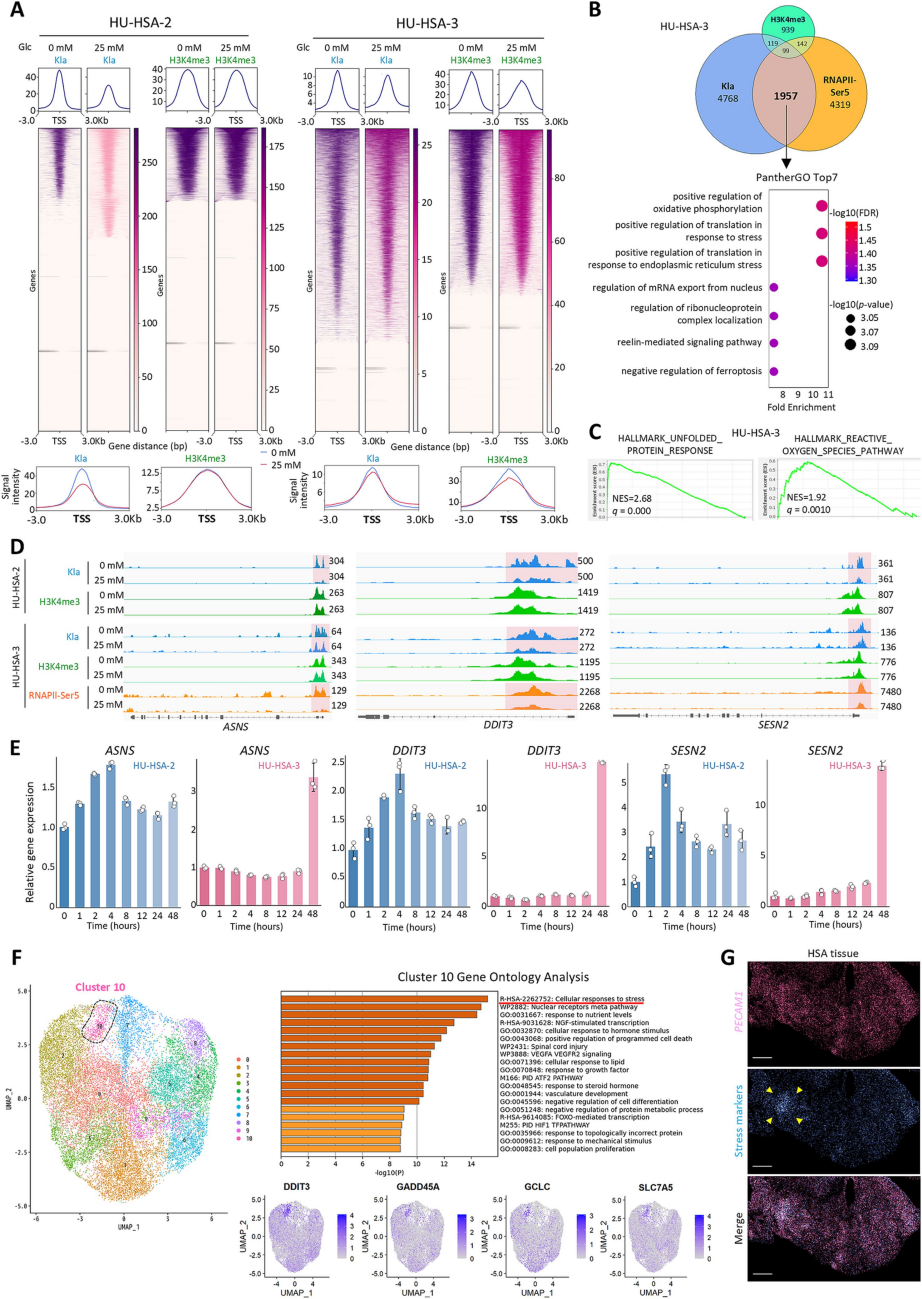
KLA is enriched at TSSs of stress-response genes and promotes their transcription under glucose starvation. **(A)** Composite profile plots (top), heatmaps (middle), and merged profile plots (bottom) around TSSs. **(B)** (Top) Venn diagram showing overlap of genes with enriched KLA, H3K4me3, or RNAPII-Ser5P at TSSs in HU-HSA-3 cells under glucose starvation. (Bottom) PANTHER gene ontology analysis of the 1,957 genes co-enriched for KLA and RNAPII-Ser5P but not H3K4me3. **(C)** GSEA plots of mRNA-seq in glucose-starved HU-HSA-3 cells. NES, normalized enrichment score. **(D)** Genome tracks showing CUT&Tag signals at representative stress-response gene loci in HSA cell lines cultured with or without glucose. **(E)** Time-course analysis of relative expression levels of stress-response genes in HSA cell lines following glucose starvation. **(F)** Integrated single-cell transcriptomic analysis of two HSA-PDX tumors. (Left) Uniform manifold approximation and projection (UMAP) of all tumor cells. (Top right) Metascape analysis for cluster 10. (Bottom right) UMAP of representative stress-response genes. **(G)** Spatial transcriptomics from an HSA patient tumor. Shown are *PECAM1* (marker of HSA tumor cells), stress-marker gene expressions, and the merged image. Arrowheads indicate a stress-response cluster. Data are presented as mean ± SD (*n* = 3).

To determine whether the robust reduction in global histone lactylation levels caused by glucose deprivation affects transcriptional regulation, we performed CUT&Tag using antibodies against lysine lactylation (Kla), H3K4me3, and H3K27ac in HU-HSA-2 cells cultured for 48 h with or without glucose. Glucose starvation increased Kla signals at promoter regions (≤ 1 kb), while the distributions of H3K4me3 and H3K27ac were not changed (Supplementary Figure S3A). Further analysis including HU-HSA-3 confirmed that Kla signals were strongly enriched around TSSs in both HU-HSA-2 and HU-HSA-3 cells under glucose-deprived conditions (Figure 2A; Supplementary Figures S3B,C). In contrast, H3K4me3 and H3K27ac did not exhibit such localized enrichment with the exception of H3K4me3 enrichment at TSSs in HU-HSA-3 (Figure 2A; Supplementary Figures S3B,C, S4A). We then analyzed co-occupancy of Kla, H3K4me3, and RNAPII-Ser5P around TSSs to assess the transcriptional competence associated with Kla signals in HU-HSA-3 cells under glucose-deprived conditions. The largest overlap (1,957 genes) was detected between Kla and RNAPII-Ser5P, whereas only 142 genes overlapped between H3K4me3 and RNAPII-Ser5P and 99 genes for all three marks, suggesting that Kla is associated with activation of gene expression independently of H3K4me3 (Figure 2B, upper). PANTHER Gene Ontology analysis of the genes overlapped with Kla and RNAPII-Ser5P identified enrichment of gene sets associated with positive regulation of OXPHOS and stress responses (Figure 2B, lower). Consistently, GSEA of mRNA-seq data showed significant enrichment of OXPHOS and stress-response signatures under glucose starvation (Figures 1I, 2C). Further examination of representative stress-response genes co-enriched with Kla and RNAPII-Ser5P at their TSSs; *ASNS* (amino-acid deprivation), *DDIT3* (endoplasmic reticulum stress), and *SESN2* (oxidative stress), confirmed concurrent enrichment under glucose-deprived conditions (Figure 2D). Expression of these and other stress-response genes was upregulated in HU-HSA-3 cells 48 h after starting glucose starvation (Figure 2E; Supplementary Figure S4B). HU-HSA-2 cells also increased expression of these genes, while transcriptional activation peaked 4 h after starting glucose deprivation (Figure 2E; Supplementary Figure S4B). Kla enrichment at TSSs was already evident 4 h after glucose withdrawal in HU-HSA-2 cells (Supplementary Figure S4C), suggesting that this cell line responds to glucose deprivation faster than HU-HSA-3 cells.

Next, to evaluate whether similar responses occur *in vivo*, we performed single-cell RNA-seq (scRNA-seq) on two HSA PDX tumors (HU-HSA-1 and HU-HSA-3) and spatial transcriptomics on an HSA patient tumor. The scRNA-seq analysis identified a tumor-cell population characterized by stress-response genes including those enriched with Kla and RNAPII-Ser5P signals at their TSSs (Figure 2F; cluster 10) of the 227 genes defining cluster 10, 44 were concurrently enriched with Kla and RNAPII-Ser5P at their TSSs (Supplementary Figure S4D). We then mapped tumor cells that expressed stress-response genes from the overlap (*ATF3, DDIT3, DDIT4, GADD45A,* and *GCLC*) as well as *ATF4* and *ASNS,* and found that they formed cluster-like regions rather than being randomly scattered, suggesting that their distribution is shaped by the local microenvironment (Figure 2G). These findings indicate that these stress-response pathways are also active *in vivo*.

Collectively, our data indicate that glucose starvation leads to Kla enrichment at TSSs of OXPHOS and stress-response genes in HSA, and that this enrichment correlates with increased transcription.

### ATF4 and acute glucose removal are required to induce stress responses

**Figure 3:**
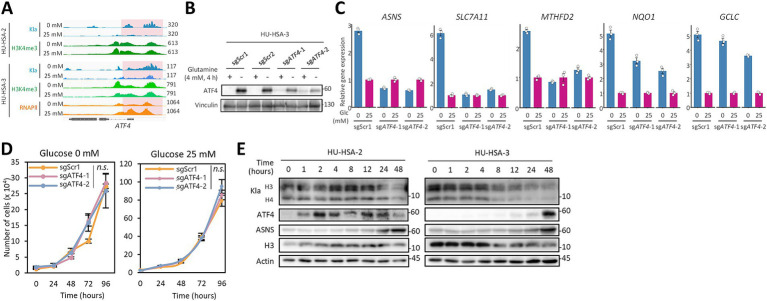
ATF4 is required to induce stress responses under glucose starvation. **(A)** Genome tracks showing CUT&Tag signals at the *ATF4* locus in HSA cell lines cultured for 48 h with or without glucose. **(B)** Western blotting of ATF4 in polyclonal ATF4-knockout HU-HSA-3 cells and scramble controls cultured without glutamine for 4 h to induce ATF4 expression. **(C)** Relative mRNA expression of key stress-response genes in scramble control and sgATF4-expressing HU-HSA-3 cells cultured for 48 h with or without glucose. Expression levels were normalized to the matched scramble control in each glucose condition. **(D)** *In vitro* growth curves of scramble control and sgATF4-expressing HU-HSA-3 cells cultured over 96 h with or without glucose. **(E)** Time course western blotting of KLa, ATF4, ASNS, total histone H3, and actin over 48 h of glucose starvation in HSA cell lines. Data are presented as mean ± SD (*n* = 3). N*.s*., not significant, two-way ANOVA in **(D)**.

**Figure 4:**
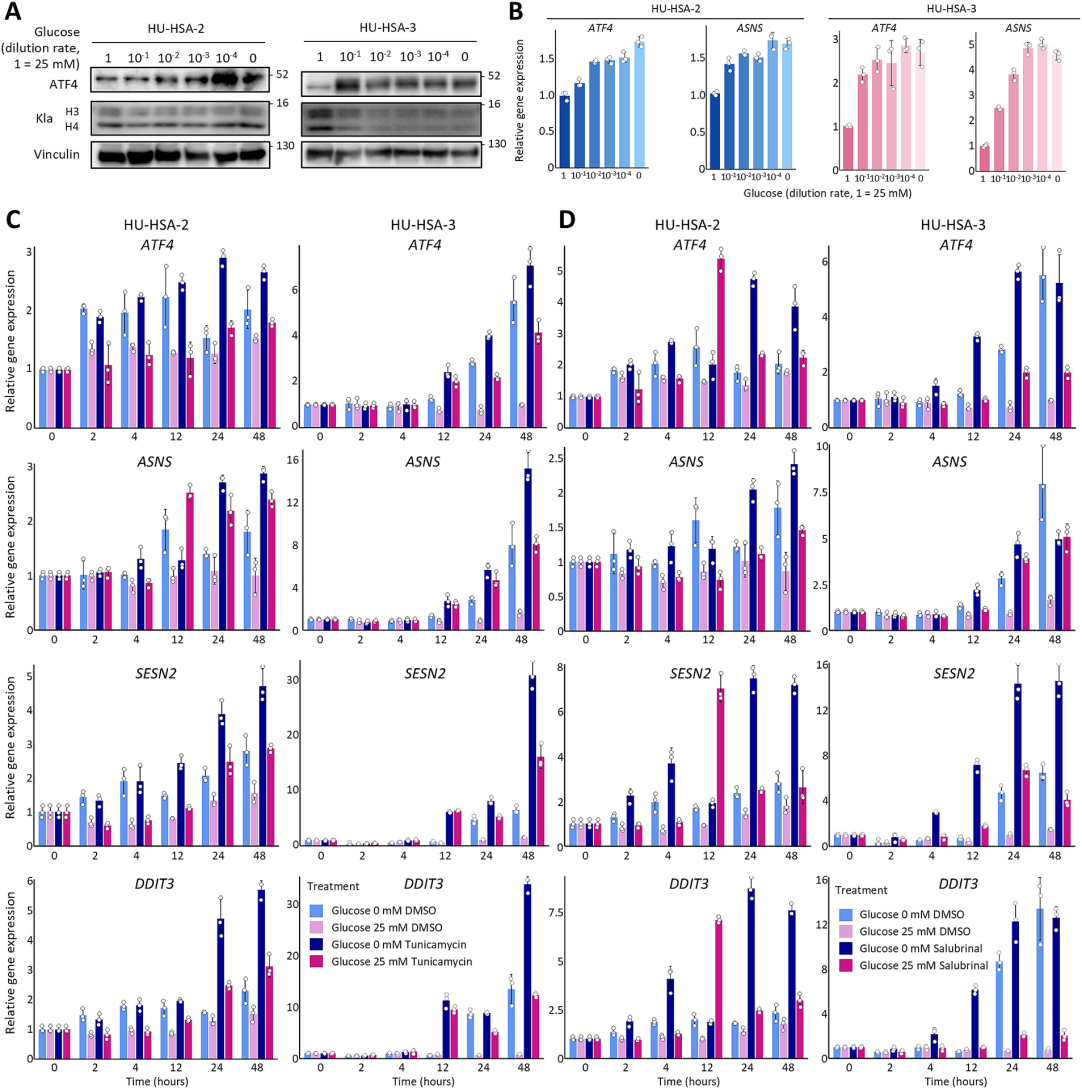
Acute glucose withdrawal is required for inducing stress responses in HSA cells. **(A)** Western blotting of ATF4, Kla, and Vinculin in HU-HSA-2 and HU-HSA-3 cells. Cells were cultured for 4 h (HU-HSA-2) or 48 h (HU-HSA-3) in medium with serially diluted glucose (25 mM to 0 mM). **(B)** Relative mRNA expression levels of *ATF4* and *ASNS* in HU-HSA-2 and HU-HSA-3 cells cultured as in **(A)**. **(C,D)** Time-course qPCR of *ATF4*, *ASNS*, *SESN2*, and *DDIT3* in HU-HSA-2 and HU-HSA-3 cells. Cells were cultured in medium with or without glucose and treated with vehicle (DMSO), 0.06 μg/mL tunicamycin **(C)**, or 10 μM salubrinal **(D)**. Data are presented as mean ± SD (*n* = 3).

To explore upstream regulation, we focused on ATF4, one of the master regulators of stress-response genes. CUT&Tag revealed robust Kla enrichment at the TSSs of ATF4 48 h after glucose withdrawal, whereas RNAPII-Ser5P enrichment was decreased (Figure 3A). Polyclonal ATF4 knockout in HU-HSA-3 cells significantly dampened the upregulation of ATF4-dependent stress-response genes induced by glucose deprivation, but it did not affect ATF4-independent stress-response genes such as *NQO1* and *GCLC* (Figures 3B,C). ATF4 loss did not affect short-term cell proliferation under either glucose-starved or normal culture conditions (Figure 3D), indicating that ATF4-regulated stress responses are negligible for short-term proliferation or that other stress-response regulators compensate for ATF4 loss. In time-course analysis of glucose deprivation, global histone lactylation levels started decreasing after 24 h in HU-HSA-2 cells and 8 h in HU-HSA-3 cells (Figure 3E). Although RNAPII-Ser5P was not enriched at TSSs of ATF4, its protein levels were increased within 1 h in HU-HSA-2 cells and by 48 h in HU-HSA-3 cells, followed by induction of ASNS (Figure 3E). Stepwise glucose dilutions indicated that ATF4 expression was induced at higher glucose concentrations in HU-HSA-3 than in HU-HSA-2 cells, although histone lactylation levels were reduced in both cell lines even at 2.5 mM glucose (10 times dilution, Figure 4A). *ATF4* and *ASNS* expressions showed inverse correlations with glucose concentrations in both cell lines; however, the extent of upregulation was greater in HU-HSA-3 cells (Figure 4B). These results suggest that HU-HSA-2 and HU-HSA-3 cell lines have different sensitivities to glucose deprivation, which could reflect their different dependencies on glucose for ATP production (Figure 1D).

We also limited glucose uptake by polyclonal knockout of GLUT1 (*SLC2A1*) and by treating cells with the GLUT1 inhibitor BAY876. Both interventions significantly reduced global histone lactylation levels (Supplementary Figures S2A, S5A), yet they failed to trigger stress-response gene expressions (Supplementary Figures S5B,C). Although BAY876 treatments slightly increased their expressions (< two-fold) in HU-HSA-2 cells, GLUT1 inhibition could be sufficient to reduce global histone lactylation levels but insufficient to induce stress responses. As noted above, long-term culture during knockout cell-line establishment might have allowed metabolic adaptation. In addition, BAY876 treatment could provide only partial inhibition, either because of incomplete inhibition of GLUT1 or compensation by other glucose transporters such as GLUT3 or GLUT4. Thus, acute and substantial glucose removal appears necessary to induce stress responses.

Next, to test whether acute glucose starvation is required for inducing stress responses, we treated the cells with tunicamycin and salubrinal. Tunicamycin directly induces endoplasmic reticulum stress by inhibiting N-linked glycosylation (59), whereas salubrinal prolongs stress responses by inhibiting eIF2α dephosphorylation, thereby increasing ATF4 protein levels (60). We used these compounds at a low-dose (25% inhibition concentration, IC_25_) to mimic glucose-deficient cultures we tested, which induced only a slight delay in cell proliferation (Supplementary Figure S5D). These treatments activated *ATF4, ASNS, SESN2,* and *DDIT3* expressions under regular culture conditions, confirming that stress responses were induced (Figures 4C,D, dark red). Glucose deprivation alone induced expression levels almost comparable to those in tunicamycin- or salubrinal-treated cells, whereas a modest additive effect was observed with drug treatments at 48 h (Figures 4C,D). In addition, these treatments induced gene expressions slightly earlier than DMSO controls in HU-HSA-3 cells, yet expression levels eventually reached similar levels by 48 h. These results suggest that acute glucose deprivation alone is sufficient to induce robust ATF4-regulated stress responses.

Taken together, glucose-starvation-induced stress responses are mostly ATF4 dependent and require acute and substantial glucose withdrawal, while loss of global histone lactylation alone is insufficient to trigger them.

### HSA cells activate *de novo* asparagine synthesis from glutamine to survive in glucose-deprived conditions

**Figure 5:**
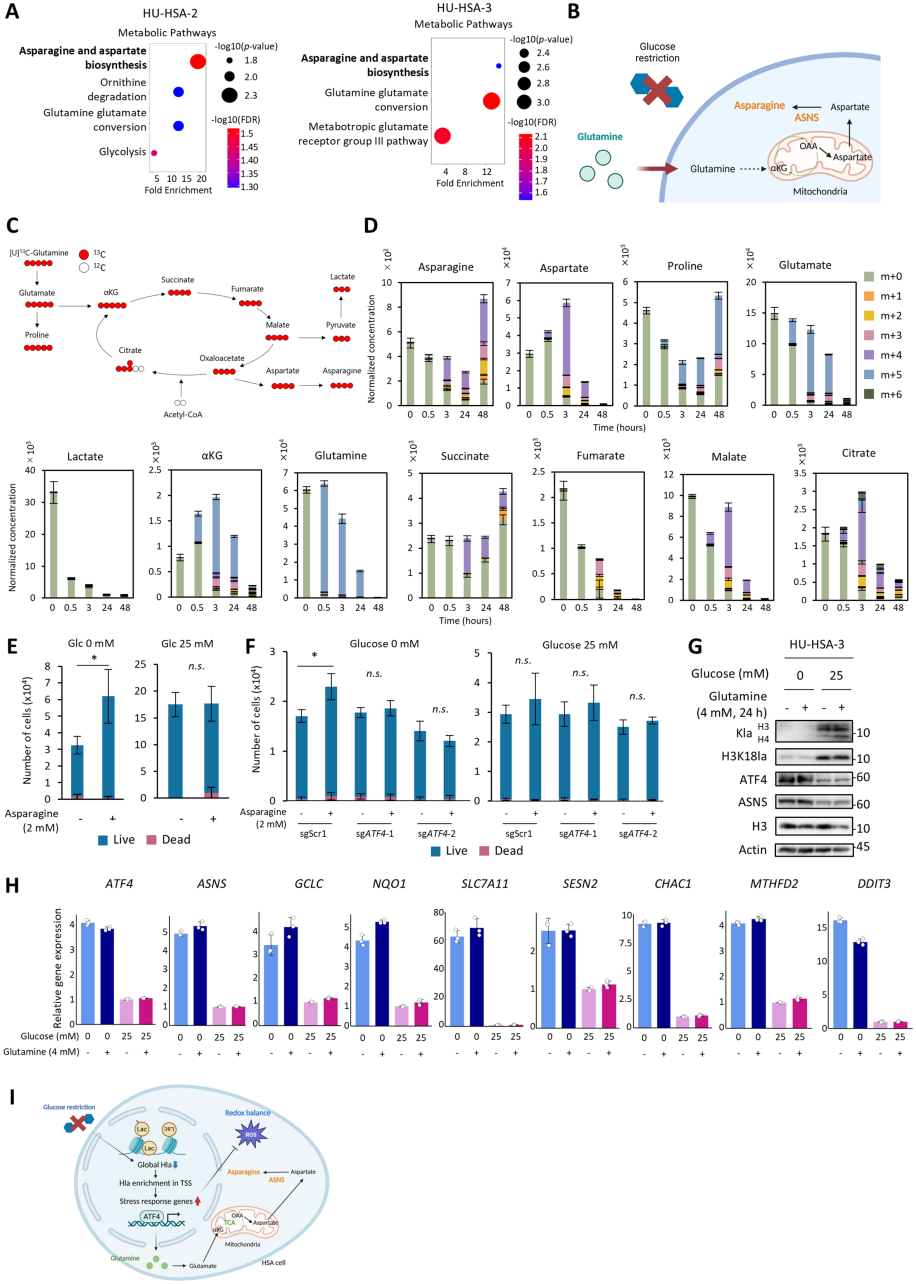
HSA cells utilize glutamine-derived asparagine for survival under glucose starvation. **(A)** PANTHER pathway analysis of genes with KLa enrichment at the TSSs in glucose-starved HSA cells. **(B)** Diagram of *de novo* asparagine synthesis via the TCA cycle under glucose restriction. Created with BioRender. **(C)** A schematic diagram of [U-^13^C]glutamine tracing. **(D)** Normalized metabolite concentrations in HU-HSA-3 cells after switching to the medium containing [U-^13^C]glutamine under glucose starvation. **(E)** Cell proliferation assay of HU-HSA-3 cells cultured for 72 h with or without glucose and supplemented with or without 2 mM asparagine in 1% FBS. **(F)** Cell proliferation assay of scramble control and sgATF4-expressing HU-HSA-3 cells cultured under the same conditions as in **(E)**. **(G)** Western blotting for KLa, H3K18La, ATF4, and ASNS in HU-HSA-3 cells treated as in **(F)**. **(H)** Relative expression levels of stress-response genes in HU-HSA-3 cells cultured for 48 h with or without glucose. 4 mM glutamine was supplemented for the final 24 h of culture. **(I)** Proposed model summarizing the adaptive response of HSA cells to glucose starvation. Created with BioRender. Data are presented as mean ± SD (*n* = 3). *p < 0.05; *n.s.*, not significant. Student’s *t* test.

So far, we have shown that glucose deprivation activates transcription of ATF4-mediated stress-response genes including the asparagine synthetase, ASNS. CUT&Tag analysis also revealed Kla enrichment at the TSSs of genes involved in asparagine and aspartate synthesis under glucose-deprived conditions (Figure 5A). We therefore hypothesized that *de novo* asparagine synthesis contributes to metabolic adaptation during acute glucose starvation. Given that glucose withdrawal increased OXPHOS activity in both cell lines and increased glutamine oxidation dependency more prominently in HU-HSA-3 (Figures 1D,H), we hypothesized that glutamine could be used for asparagine synthesis via anaplerosis (Figure 5B).

To trace glutamine-derived carbon, we conducted isotope-tracing metabolomic analysis using [U-^13^C]glutamine for 48 h in HU-HSA-3 cells (Figure 5C). The results confirmed that glutamine fueled the tricarboxylic acid (TCA) cycles since ^13^C labeling appeared in TCA-cycle intermediates within 0.5 h (Figure 5D). ^13^C incorporation into asparagine started 3 h after glucose starvation, and asparagine concentration significantly increased by 48 h (Figure 5D). By contrast, ^13^C enrichment and the concentration of aspartate peaked at 3 h and then decreased gradually, suggesting conversion of aspartate to asparagine by ASNS. Lactate concentration was markedly reduced 0.5 h after glucose starvation (Figure 5D). This confirms that Kla enrichment at TSSs was established under low-lactate conditions. Next, we added asparagine (2 mM at a final concentration) to the culture medium under glucose-starved and low-FBS conditions. In this experiment, we reduced FBS concentrations to minimize the effect of serum-derived asparagine and to examine the direct effect of asparagine supplementation. Asparagine supplementation modestly increased HSA cell proliferation rates under glucose-deprived conditions for 72 h (Figure 5E; Supplementary Figure S6A), and this effect was abolished by ATF4 reduction (Figure 5F). In contrast, asparagine supplementation had no impact under normal glucose conditions (Figure 5E; Supplementary Figure S6A). Although proline concentration showed a similar trend to that of asparagine (Figure 5D), proline supplementation did not accelerate HSA cell proliferation, nor were proline-synthesis genes upregulated (Supplementary Figures S6B,C). These results suggest that glutamine-derived asparagine, produced through the ATF4-ASNS axis, supports HSA cell survival in glucose-deprived conditions.

^13^C tracing also indicated that intracellular glutamine was significantly decreased at 24 h and was nearly depleted at 48 h (Figure 5D), which raised a possibility that glutamine deficiency, rather than glucose starvation, induced stress responses. To address this possibility, we added glutamine 24 h after initiation of glucose starvation and subsequently examined stress-response gene and protein expression. Glutamine supplementation did not affect gene expression changes induced by glucose starvation, global histone lactylation levels, and ATF4/ASNS protein expression levels (Figures 5G,H). These results suggest that these responses are driven primarily by glucose withdrawal rather than by secondary glutamine depletion during the extended culture period.

Collectively, we demonstrate that HSA cells activate *de novo* asparagine synthesis from glutamine to adapt to glucose starvation, and that ATF4-mediated stress responses and global histone lactylation loss are induced by glucose deprivation itself (Figure 5I).

### M2–like macrophages accumulate around HSA cells with low histone lactylation levels

**Figure 6:**
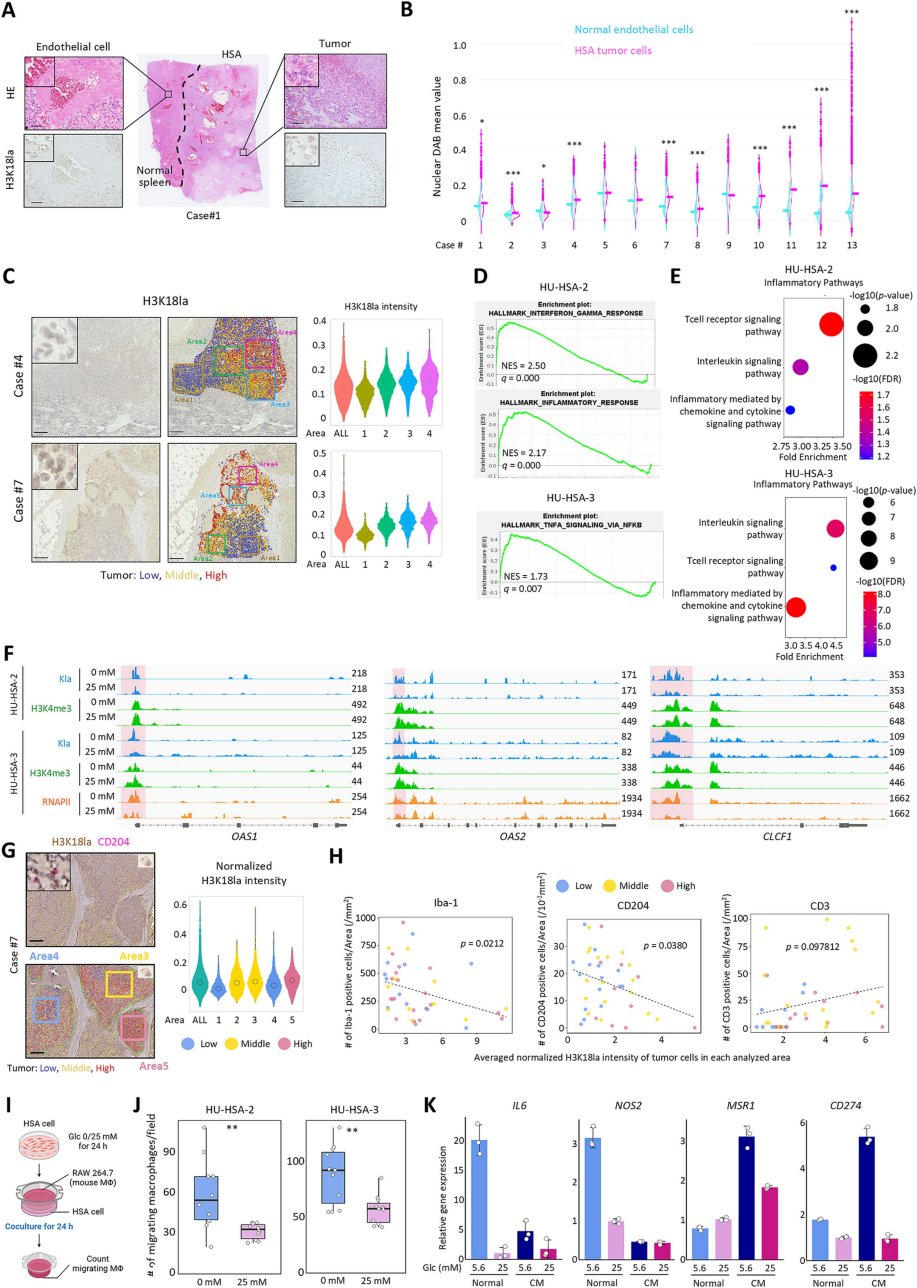
Histone lactylation exhibits heterogeneous distribution, and M2-like macrophages accumulate in low-histone lactylation areas. **(A)** Representative images of hematoxylin & eosin and H3K18la IHC of HSA and adjacent normal tissues. Scale bars, 100 μm. **(B)** Quantitative analysis of nuclear H3K18la intensity in HSA and normal endothelial cells. **(C)** (Left) Representative images showing intratumoral H3K18la heterogeneity in HSA tissue. Scale bars, 250 μm. (Right) Violin plots showing H3K18la intensity for total and subregions. **(D)** GSEA plots from mRNA-seq showing enrichment of inflammatory pathways in glucose-starved HSA cells. **(E)** PANTHER pathway analysis of Kla-enriched genes from glucose-starved HSA cells. **(F)** IGV snapshots of CUT&Tag signals at representative inflammatory gene loci. **(G)** Representative dual-IHC images for H3K18la and CD204 in HSA tissue. Scale bars, 200 μm. **(H)** Correlations between tumor-cell H3K18la intensity and the number of infiltrating immune cells in each area. **(I)** Schematic of the migration assay. **(J)** Box plots of migrating RAW264 cells. **(K)** Relative mRNA expression levels in RAW264 cells treated with conditioned medium from HU-HSA-3 cells cultured with or without glucose. Data are presented as mean ± SD (*n* = 3). *p < 0.05, **p < 0.01, ***p < 0.001. Student’s *t* test.

Finally, we examined histone lactylation levels and its functional implications in patient tumors. Formalin-fixed paraffin-embedded blocks from 13 canine splenic HSA cases archived in our laboratory were used for this purpose (Table 3). Immunohistochemistry (IHC) for H3K18la indicated that HSA tumor cells exhibited significantly higher average H3K18la intensity compared to normal endothelial cells (ECs) in 9 out of 13 cases (Figures 6A,B). In contrast, no such trend was observed in nuclear Kla signals likely because this antibody recognizes broader targets including non-histone proteins (Supplementary Figures S7A,B). By careful microscopic observation, we recognized heterogeneous H3K18la signal patterns among tumor cells within the same tissues. We then classified tumor cells into low, middle, and high groups based on nuclear H3K18la mean values and visualized their spatial distribution. The results indicated that tumor cells formed clusters with cells of the same group, suggesting that H3K18la levels are associated with spatial factors such as the tumor microenvironment (Figure 6C).

mRNA-seq and CUT&Tag experiments on glucose-deprived HSA cell lines revealed transcriptional activation and Kla enrichment at the TSSs of inflammation-associated genes (Figures 6D–F). Based on these findings, we double-stained H3K18la and immune-cell markers (Iba-1 for macrophages, CD204 for M2-like macrophages, and CD3 for T cells) to evaluate correlations between histone lactylation and immune responses. To take heterogeneous H3K18la patterns into account, we first classified tumor cells as described above and selected five areas that included all three groups for each tissue sample (Figure 6G). The results indicated statistically significant negative correlation between average normalized H3K18la signals and Iba-1-positive or CD204-positive cells (*p* = 0.0212 and *p* = 0.0380, respectively), whereas no correlation was observed with CD3-positive cells (Figure 6H). These findings suggest that macrophages, particularly those with an M2-like phenotype, preferentially infiltrate into tumor regions characterized by low histone lactylation levels.

To further explore functional interactions between glucose-starved HSA cells and macrophages, we co-cultured HSA cells with murine macrophage cell line RAW264 (Figure 6I). Glucose-starved HSA cells attracted a significantly higher number of RAW264 cells compared to HSA cells cultured under regular glucose conditions (Figure 6J). Furthermore, conditioned medium from glucose-restricted HSA cells decreased expression of M1-like markers (*IL-6* and *NOS2*) and increased expression of M2-like markers (*MSR1* and *CD274*) in RAW264 cells (Figure 6K). These results indicate that glucose-starved HSA cells attract macrophages and polarize them toward an M2-like phenotype.

Taken together, we demonstrate that histone lactylation is spatially heterogeneous in HSA tissues, and that tumor regions with low histone lactylation levels recruit M2-type macrophages. Considering these findings together with the *in vitro* co-culture experiment results, HSA cells can create pro-tumor microenvironments in glucose-restricted regions in tumor tissues.

## Discussion

In this study, glucose withdrawal from the culture medium resulted in a redistribution of Kla to TSSs of genes associated with stress responses, asparagine synthesis, and immune responses. Stress-response genes exhibited concomitant enrichment of Kla and RNAPII-Ser5P at TSSs and were transcriptionally upregulated, suggesting that Kla at TSSs is associated with positive regulation of transcription. This is consistent with previous findings that histone lactylation is enriched at TSSs and actively regulates gene expression in tumor cells and immune cells (7, 61, 62). These studies, however, were conducted under glucose-rich conditions. Our results indicate that HSA cells exploit a limited lactate pool to regulate specific genes under glucose- and lactate-restricted conditions, suggesting that tumor cells can rapidly adapt to microenvironmental changes. Although we identified the important role of Kla under glucose-deprived conditions, we could not determine whether enrichment at TSSs was established on histones. These signals may reflect lactylation of histones, non-histone proteins, or both. We tested an H3K18la antibody in CUT&Tag as reported elsewhere but failed to obtain sufficient DNA for sequencing. This might be attributed to insufficient H3K18la levels under glucose-deprived conditions, lot-to-lot variability of the antibody, or species differences. Nevertheless, the observed redistribution of Kla under glucose-deprived conditions suggests that HSA cells utilize a limited lactate pool to epigenetically regulate gene expression and adapt to low-glucose environments.

HU-HSA-2 and HU-HSA-3 differed in the timing and magnitude of transcriptional stress responses to glucose starvation, consistent with intrinsic metabolic heterogeneity. Our mitochondrial fuel-usage profiling and transcriptomic analyses suggest that HU-HSA-3 is more metabolically flexible and more readily engages oxidative metabolism and alternative substrates (e.g., fatty acids and glutamine) when glucose becomes limiting, which would be expected to buffer early energetic stress and delay ATF4-dependent transcriptional activation relative to HU-HSA-2. In addition, HU-HSA-3 shows higher basal histone lactylation under glucose-replete conditions, which may provide a chromatin-level reserve that supports a more gradual transition during nutrient restriction, whereas lower basal lactylation levels in HU-HSA-2 may contribute to a faster onset of stress signaling. Together, these cell line–dependent features likely reflect broader metabolic diversity in HSA and may help explain variable adaptation to glucose-poor microenvironments.

Although genes associated with asparagine synthesis and immune responses showed Kla peaks at their TSSs, they were not overrepresented among PANTHER GO terms for genes showing concomitant enrichment of Kla and RNAPII-Ser5P. This means that genes with Kla peaks at their TSSs do not necessarily coincide with RNAPII-Ser5P peaks, although some genes such as *OAS1*, *OAS2*, and *CLCF1* exhibited co-enrichment of both signals. We speculate that these lactylation marks prime specific genes for rapid expression upon a secondary stimulus. Several reports indicate similar mechanisms in innate immunity. M1 macrophages exposed to bacteria primed wound-healing genes that were later transcribed during M2 polarization, and trained monocytes/macrophages retain H3K18la as an epigenetic memory that accelerates gene expression upon a secondary stimulus (7, 63). While our experiments demonstrated that the asparagine pathway and immune responses are functionally important in HSA cells, time-course analyses of gene and protein expression will be required to determine whether Kla actually primes these genes for rapid reactivation upon a secondary stimulus. In addition, our CUT&Tag experiments revealed that ATF4 was one of the genes with Kla enrichment at their TSSs without RNAPII-Ser5P enrichment. Given that ATF4 protein expression is regulated by translational inhibition (64), concomitant enrichment of Kla and RNAPII-Ser5P may not be necessary for immediate induction. However, it is possible that Kla at TSSs functions as an epigenetic memory to rapidly supply additional ATF4 protein if its levels become limiting or additional cellular stresses occur. Overall, although further research is required, Kla, possibly histone lactylation, could maintain selected genes in a poised state for future stimuli in tumor cells as well.

In this study, we established and characterized canine HSA cell lines and PDX models by examining their morphology, gene and protein expression, and STR profiles. Long-term *in vitro* cultures can induce genetic drift, clonal selection, and altered signaling-pathway activity, thereby causing tumor cells to lose their original characteristics (65, 66). This makes it difficult to predict patient responses (67, 68). To minimize this limitation, in our study, all *in vitro* experiments were conducted with early-passage cultures (fewer than p16). In addition, PDX models are useful tools to predict patient responses to potential therapeutics. Generally, PDX models retain morphology and heterogeneity more faithfully than cultured cells because they are grown in a 3D environment with non-tumor components such as stromal and immune cells (69). Indeed, our HSA PDX models recapitulated original patient tumor morphology more accurately than early-passage cell lines. Although further characterization is needed, our paired patient-derived HSA models could be useful for basic and translational hemangiosarcoma research.

Our study has several limitations. First, all metabolic analyses were performed in 2D cell cultures. Tumor-cell metabolism *in vivo* is modulated by microenvironmental factors, which results in metabolic dynamics that differ from those *in vitro*. Future studies should interrogate HSA cell metabolism in cell-line xenografts, PDX models, and patient tissues for a more physiologically relevant understanding. Second, we could not perform loss- and gain-of-function experiments targeting histone lactylation. Writers and erasers for histone lactylation have been identified, but they can also modify histone acetylation (7, 70). This means that their knockout, overexpression, or pharmacological inhibition can affect multiple histone modifications, which obscures whether Kla peaks at TSSs drive transcription or merely correlate with a transcriptionally active epigenetic state. Third, our experiments were limited to canine hemangiosarcoma. Whether the glucose-deprivation-associated enrichment of pan-Kla at TSSs is conserved in human angiosarcoma or across other tumor types remains unknown. If conserved, this lactylation-based gene regulation could be targeted broadly. If not, it may be unique to HSA and should be pursued as an HSA-specific target. Nonetheless, our data show that lysine lactylation, possibly histone lactylation, persists even under glucose-deprived conditions, suggesting that tumor cells exploit this epigenetic mark to regulate transcription under nutrient-poor conditions. Fourth, we used the murine macrophage cell line RAW264 for co-culture and conditioned medium assays due to the limited availability of canine macrophage cell lines. Although RAW264 cells are widely used as a macrophage model, interspecies differences in cytokine-receptor affinity or signal interaction could affect the results. Future studies using canine-derived macrophages are warranted to validate the species-specific interactions observed in this study.

## Data Availability

The datasets generated and/or analyzed during the current study are available from the corresponding author on reasonable request. mRNA-seq and CUT&Tag data were uploaded on Gene Expression Omnibus (GSE304507, GSE304509, and GSE305701). Custom codes used for data analysis are available at Zenodo (DOI: 10.5281/zenodo.16813072). All noncommercially available new materials, including constructs, cell lines and PDX models, that Hokkaido University has the right to provide will be made available to nonprofit or academic requesters upon completion of a standard material transfer agreement. Requests for materials may be made by contacting KA (k-aoshima@vetmed.hokudai.ac.jp).

## Footnotes

1
https://horizondiscovery.com/ja/ordering-and-calculation-tools/crispr-design-tool

2
http://crispor.tefor.net/

3
https://crispr.dbcls.jp/
