# Supplementary material for: Lysine lactylation regulates ATF4-mediated stress responses under glucose starvation in canine hemangiosarcoma
Source: Front Vet Sci. 2026 Feb 12;13:1734339. doi: 10.3389/fvets.2026.1734339 (PMC12936438; doi:10.3389/fvets.2026.1734339)
Supplement: Supplementary file 1 [file Data_Sheet_1.pdf]

Supplementary Materials for

Lysine lactylation regulates ATF4-mediated stress responses under glucose starvation in canine hemangiosarcoma

Tamami Suzuki *et al.*

Corresponding author: Keisuke Aoshima, k-aoshima@vetmed.hokudai.ac.jp

The PDF file includes:

Figs. S1 to S7

Supplementary Figure. 1

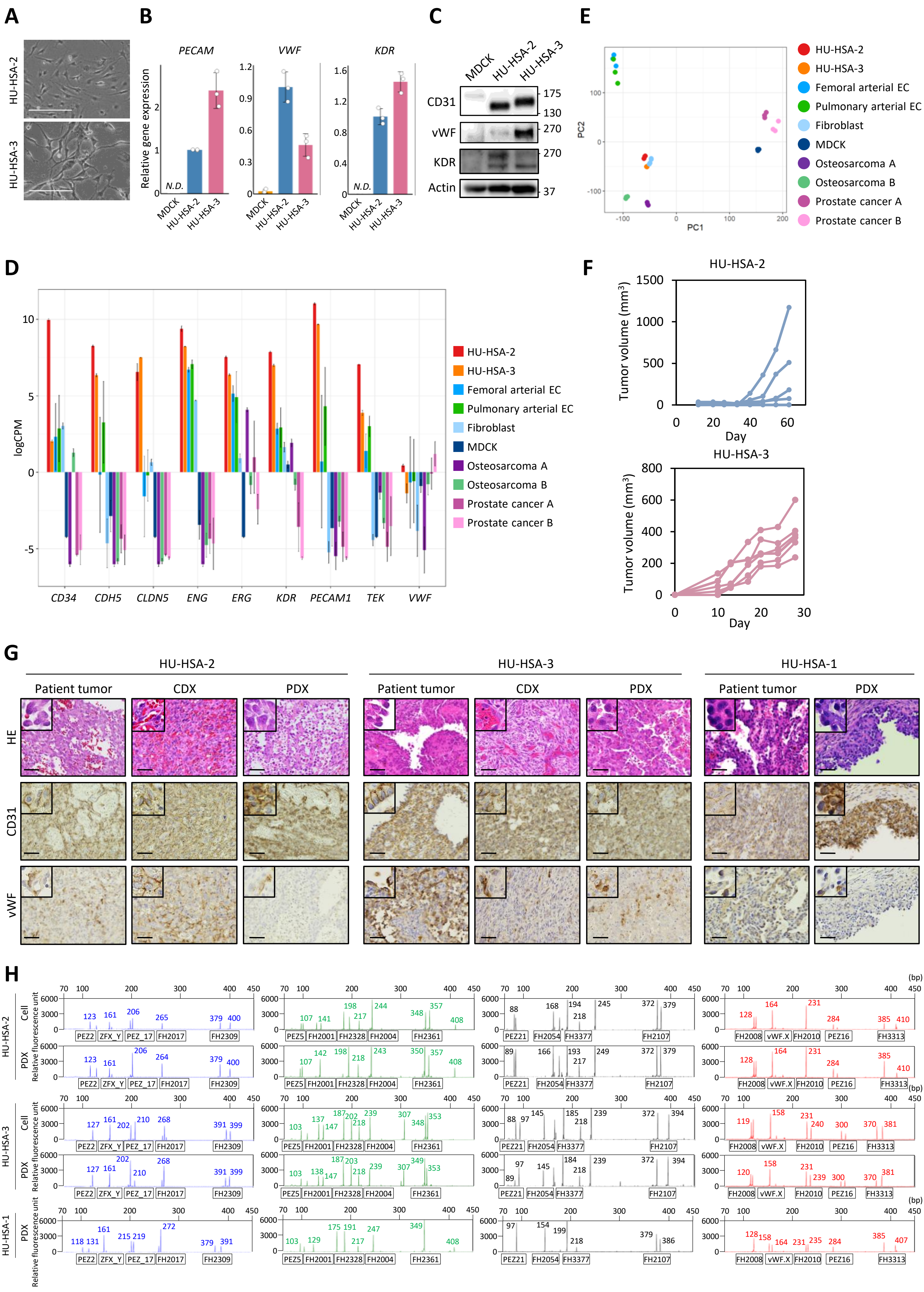

Fig. S1. Established HSA cell lines and PDX models retained HSA features.

(A) Phase-contrast images of established HSA cell lines. Scale bars, 250 μm. **(B)** Relative expression levels of endothelial marker genes in HU-HSA-2 and HU-HSA-3 cells with MDCK cells as the negative control. **(C)** Western blot analysis of endothelial marker proteins in HU-HSA-2 and HU-HSA-3 cells. MDCK cells were used as a negative control. **(D)** Principal component analysis of RNA-seq data from HSA cell lines and canine tumor or healthy cells. **(E)** mRNA expression levels of endothelial marker genes in HSA cell lines and canine tumor or normal cells. **(F)** Tumor growth curves of HU-HSA-2 and HU-HSA-3 cells transplanted into nude mice. Each line represents the volume of tumors formed in individual injection sites (n = 6 injection sites; two per mouse). Given that tumors did not develop at two of the HU-HSA-2 injection sites, the corresponding growth curves overlap along the x-axis. **(G)** Representative images of H&E staining and IHC for the endothelial markers. Images compare the original patient tumor, cell line-derived xenograft (CDX), and PDX for both HU-HSA-2 and HU-HSA-3, and the patient tumor and PDX for HU-HSA-1. Scale bars, 50 μm. **(H)** STR profiles of the established cell lines and their corresponding PDX models. Data are presented as average ± SD from three technical replicates. *N.D.*, not detected.

Supplementary Figure. 2

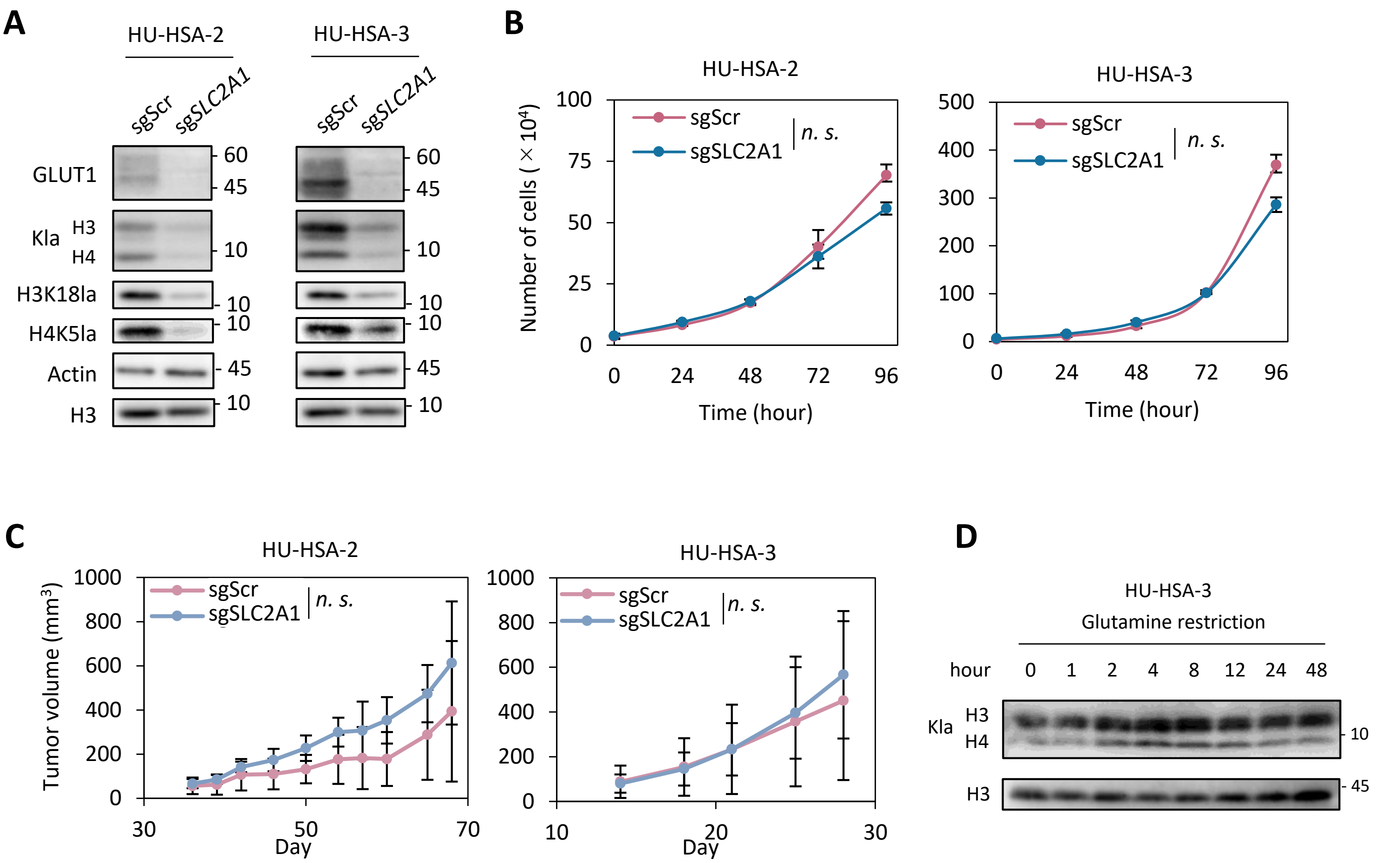

Fig. S2. *SLC2A1* polyclonal knockout decreases global histone lactylation levels but does not significantly affect HSA cell growth.

(A) Western blot analysis to assess GLUT1 suppression efficiency and global Kla, H3K18la, and H4K5la levels in HSA cell lines expressing the sgScramble (sgScr) and sgSLC2A1. **(B)** *In vitro* growth curves of HSA cell lines expressing sgScr and sgSLC2A1 over 96 hours. Data are presented as mean ± SD (three biological replicates). **(C)** *In vivo* tumor growth curves of HSA cell lines expressing sgScr and sgSLC2A1 transplanted subcutaneously into each flank of nude mice. Data are presented as mean ± SD ($n = 6$ tumors per group). **(D)** Time-course western blotting of Kla in HU-HSA-3 cells cultured in glutamine-free medium for 0 - 48 hours.
n.s., not significant. Two-way ANOVA.

Supplementary Figure. 3

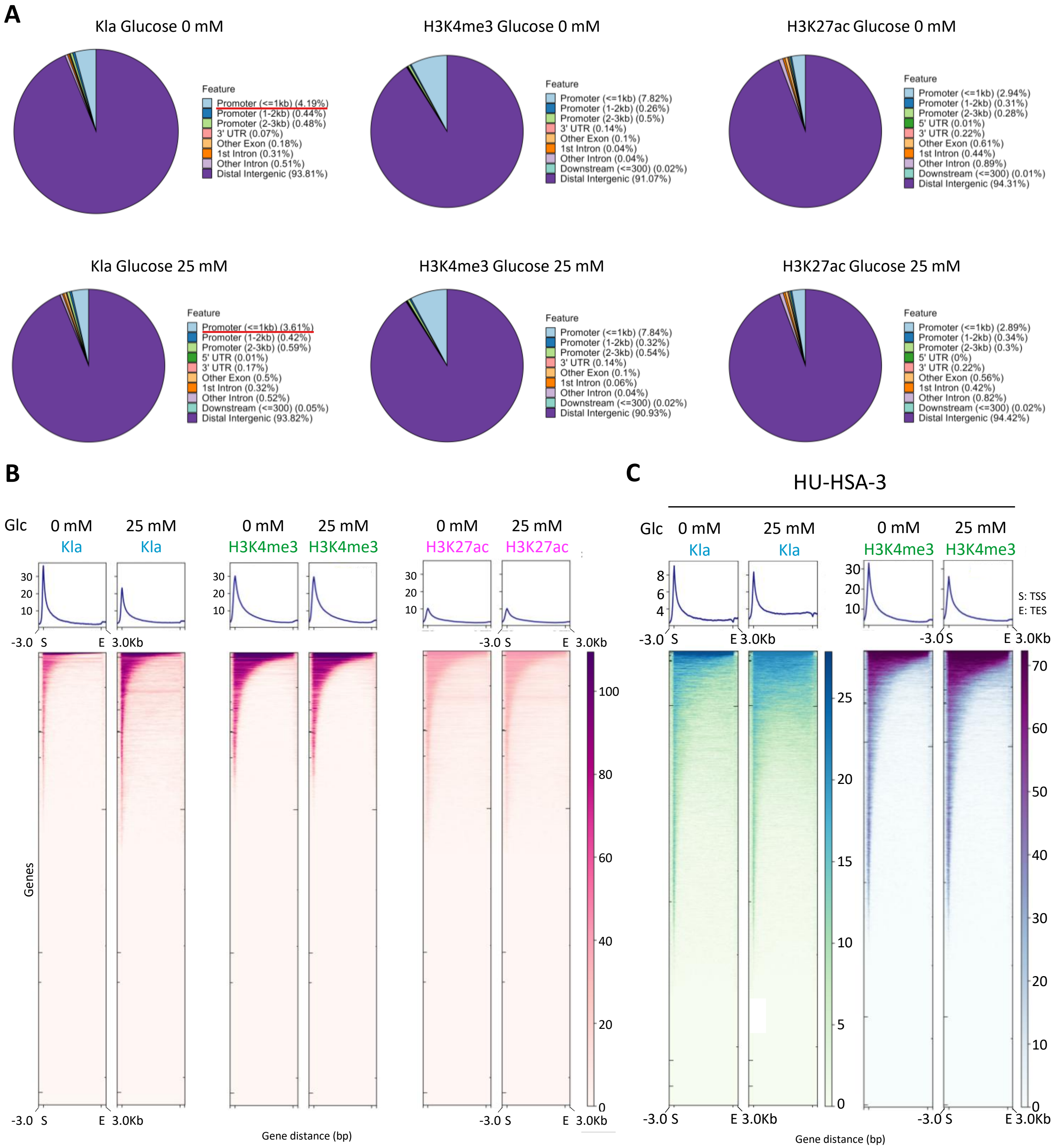

Fig. S3. Kla is enriched around TSSs under glucose starvation in HSA cells.

(A) Pie charts showing the genomic distribution of Kla, H3K4me3, and H3K27ac peaks obtained from CUT&Tag analysis in HU-HSA-2 cells cultured with or without glucose for 48 hours. **(B, C)** Composite profile plots (top) and heatmaps (bottom) on gene bodies showing the distribution of Kla, H3K4me3, and H3K27ac enrichment in HU-HSA-2 (B) and HU-HSA-3 (C) cells cultured with or without glucose for 48 hours.

Supplementary Figure. 4

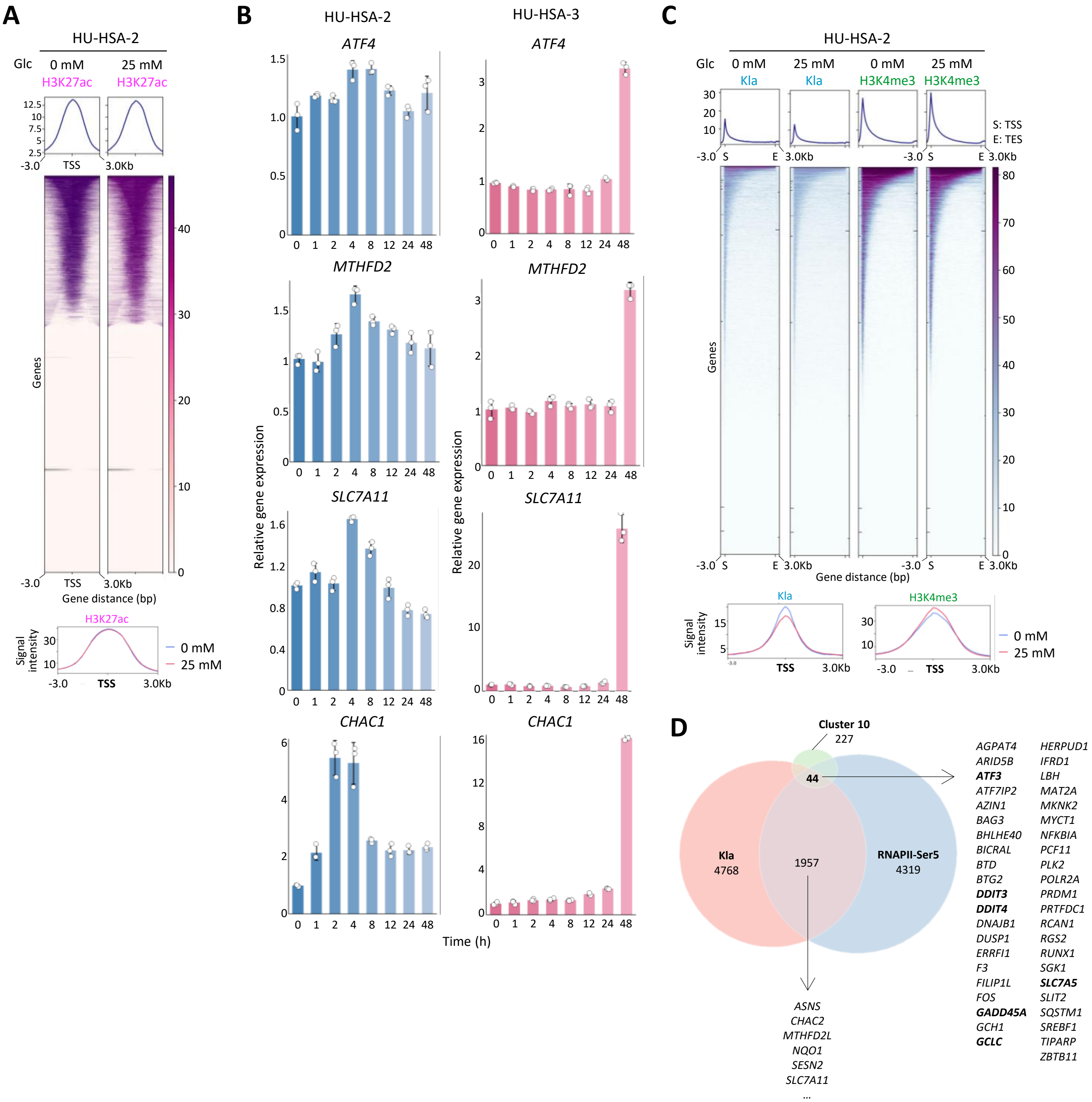

Fig. S4. Kla is enriched around TSSs of stress-response genes under glucose starvation in HSA cells.

(A) Composite profile plots (top), heatmaps (middle), and merged profile plots (bottom) around TSSs showing the distribution of H3K27ac enrichment in HU-HSA-2 cells cultured with or without glucose for 48 hours. **(B)** Time-course relative expression levels of *ATF4* and selected target genes in HSA cell lines after glucose starvation. Data are presented as mean ± SD from three technical replicates. **(C)** Composite profile plots (top), heatmaps (middle), and profile plots around TSSs (bottom) showing the distribution of Kla and H3K4me3 enrichment in HU-HSA-2 cells cultured with or without glucose for 4 hours. **(D)** Venn diagram showing overlap between cluster 10 marker genes from scRNA-seq and genes with Kla and RNAPII-Ser5 enrichment at TSSs in HU-HSA-3 cells after glucose starvation. 44 of Kla and RNAP-Ser5 enriched genes were cluster 10 markers (listed at right).

Supplementary Figure. 5

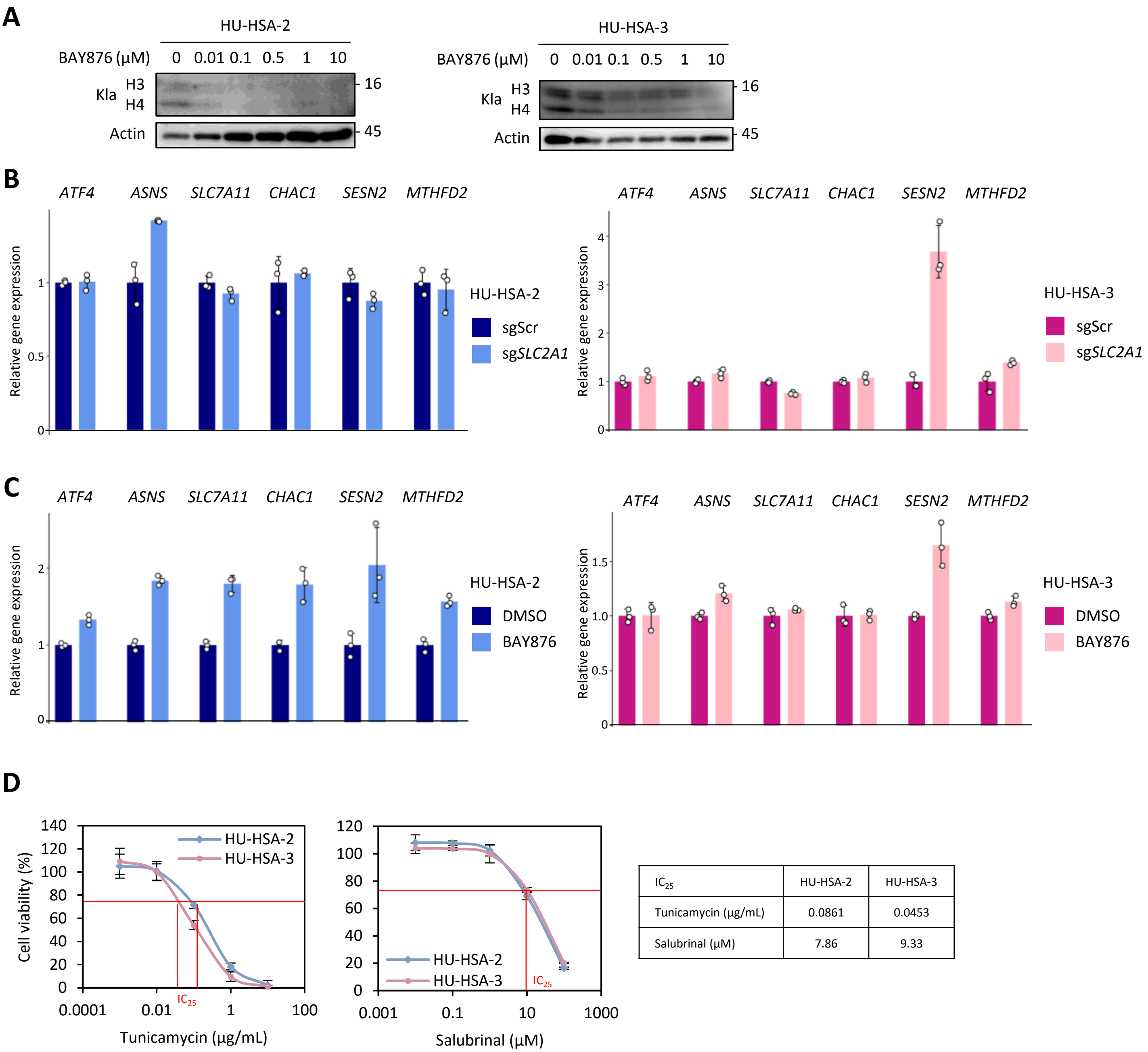

IC25	HU-HSA-2	HU-HSA-3
Tunicamycin (μg/mL)	0.0861	0.0453
Salubrinal (μM)	7.86	9.33

Fig. S5 GLUT1 inhibition reduces global histone lactylation but fails to activate stress-response genes.

(A) Western blot analysis for Kla in HSA cell lines treated with BAY876 (0 - 10 μM) for 48 hours. **(B, C)** Relative expression levels of key stress-response genes in HSA cell lines expressing sgScr or sgSLC2A1 (B), or treated with either DMSO or 0.1 μM BAY876 (C). Data are presented as average ± SD from three technical replicates. Gene expressions were normalized to that of the corresponding controls. **(D)** Cell viability curves of HU-HSA-2 and HU-HSA-3 cells treated with tunicamycin or salubrinal for 48 hours. Red lines indicate the line for 75% survival. Data are presented as average ± SD from three technical replicates.

Supplementary Figure. 6

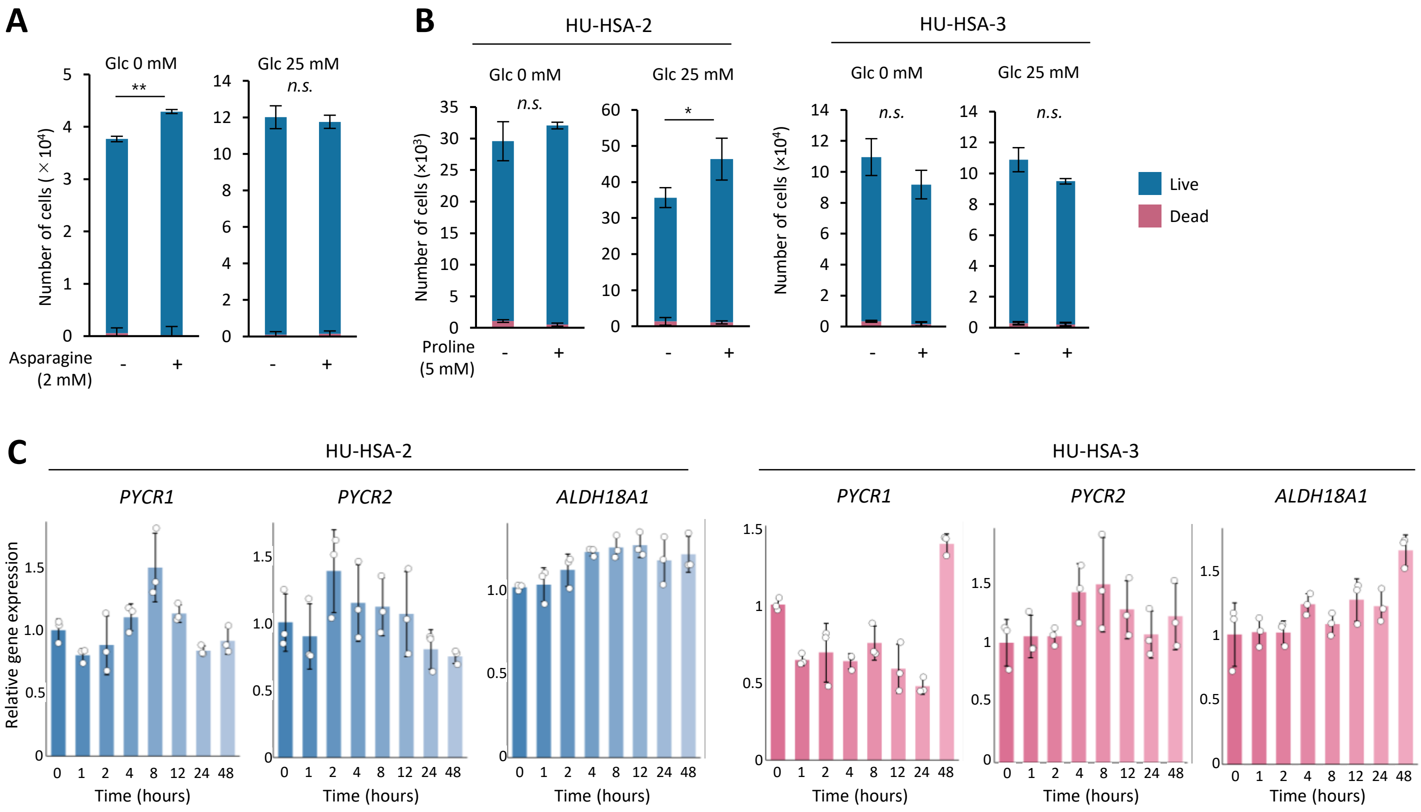

Fig. S6 Asparagine modestly accelerates HSA cell proliferation, while proline does not.

(A) The number of HU-HSA-2 cells cultured for 72 hours in regular or glucose-free medium supplemented with 2 mM asparagine and 3% FBS. Data are presented as average ± SD from three biological replicates. $^{**}P < 0.01$; n.s., not significant; Student's *t* test. **(B)** The number of HU-HSA-2 and HU-HSA-3 cells cultured for 72 hours in regular or glucose-free medium supplemented with 5 mM proline and 1% FBS. Data are presented as average ± SD from three biological replicates. $^{*}P < 0.05$; n.s., not significant; Student's *t* test. **(C)** Time-course relative expression levels of proline synthesis-related genes in HU-HSA-2 and HU-HSA-3 cells following glucose starvation. Data are presented as average ± SD from three technical replicates.

Supplementary Figure. 7

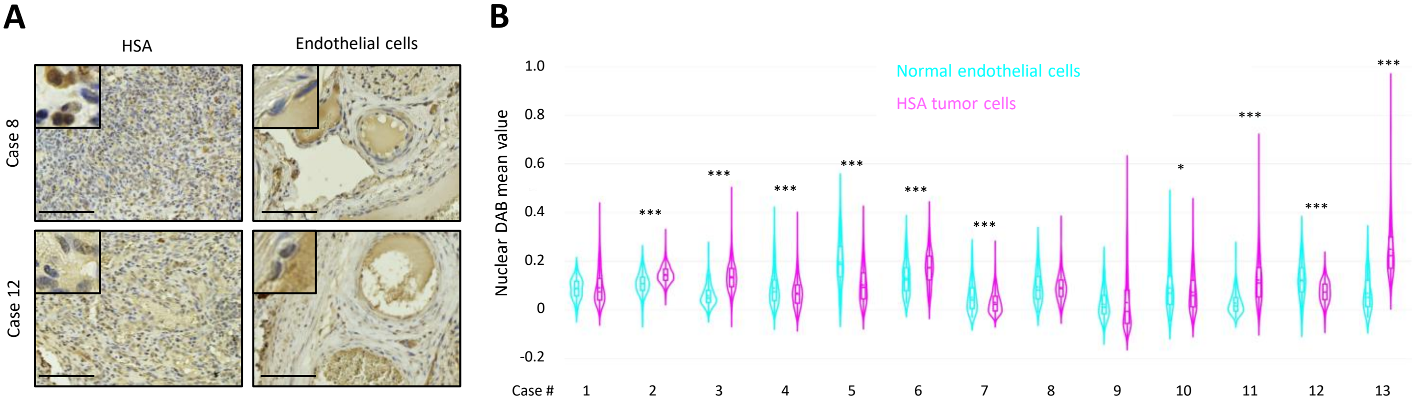

Fig. S7 IHC analysis for Kla shows no consistent trend between HSA cells and normal endothelial cells.

(A) Representative images of Kla staining in HSA tissues and adjacent normal splenic tissues. Scale bars, 100 µm. **(B)** Violin plots of nuclear pan-Kla intensities in normal endothelial cells versus HSA tumor cells across 13 patient cases. ***$P < 0.001$, *$P < 0.05$. Student's t test.
